# Statistical modeling for COVID 19 infected patient’s data in Kingdom of Saudi Arabia

**DOI:** 10.1371/journal.pone.0276688

**Published:** 2022-10-28

**Authors:** Ramy Aldallal, Ahmed M. Gemeay, Eslam Hussam, Mutua Kilai

**Affiliations:** 1 Department of Accounting, College of Business Administration in Hawtat bani Tamim, Prince Sattam bin Abdulaziz University, Al-Kharj, Saudi Arabia; 2 Department of Mathematics, Faculty of Science, Tanta University, Tanta, Egypt; 3 Department of Mathematics, Helwan University Faculty of Science, Helwan, Egypt; 4 Pan African Institute of Basic Science, Technology and Innovation, Nairobi, Kenya; Amity University - Lucknow Campus, INDIA

## Abstract

The objective of this study is to construct a new distribution known as the weighted Burr–Hatke distribution (WBHD). The PDF and CDF of the WBHD are derived in a closed form. Moments, incomplete moments, and the quantile function of the proposed distribution are derived mathematically. Eleven estimate techniques for estimating the distribution parameters are discussed, and numerical simulations are utilised to evaluate the various approaches using partial and overall rankings. According to the findings of this study, it is recommended that the maximum product of spacing (MPSE) estimator of the WBHD is the best estimator according to overall rank table. The actuarial measurements were derived to the suggested distribution. By contrasting the WBHD with other competitive distributions using two different actual data sets collected from the COVID-19 mortality rates, we show the importance and flexibility of the WBHD.

## 1 Introduction

The creation of effective statistical models for natural and real-life occurrences that may be represented by established statistical probability distributions is one of the fundamental goals of statistics. Where the probability distributions are being utilised to simulate the unpredictable and potentially dangerous life occurrence that is of interest to the researcher. Because of the complexity and difficulties involved in simulating real life occurrences using standard distributions, a large number of other probability distributions have been devised.

Sometimes, the known and accessible probability distributions continue to be unable to adequately reflect and describe the facts for particular natural events. This may be frustrating. The generalised probability distributions are the ones that end up being expanded and modified as a consequence of these changes and expansions. For more readings see [1].

The addition of a few new or additional parameters to well-known probability distributions improved the applicability of those distributions for the data pertaining to natural events and raised the accuracy with which they presented the tail shape of the distribution. There are various helpful methods to expand and increase the flexibility of the traditional statistical distributions. One of these ways is by including an extra parameter in the distribution. One example of this is the power (P) transformation.

In this article, the two-parameter WBHD, which has a variety of interesting traits, is obtained by referring to the distributions discussed earlier in the subject. Because it may be skewed to the right as positive skewed, skewed to the left as negative skewed, or symmetric, the implemented WBHD does have a PDF that is more flexible. This provides for extra tail flexibility. It can mimic decreasing, rising, bathtub, and reverse-J hazard rates as well as other hazard rate scenarios. In addition to that, the distribution that has been proposed has an exact closed-form CDF and PDF can be managed with relative ease. Because of these benefits, the distribution has a promising potential for applications in a wide range of industries, such as biotechnological life testing, durability, and econometric data. For more readings see [2–7].

Recent years have seen an uptick in the number of writers interested in developing novel lifetime distributions for the purpose of fitting actual lifetime data. One of them is: [8–16]. On the other hand, it is common knowledge that order statistics may deal and apply with the applications and attributes of random variables and of functions associated to them see [17–19] for reference.

Whether we need to have this distribution is the most important issue. In order to answer this question, we will briefly summarise the relevance of the WBHD: (i) The statistical functions of the WBHD may be expressed in a straightforward and closed-form manner. (ii) the features of the WBHD may be inferred clearly without the need of any special and particular mathematical functions; and (iii) the proposed WBHD provides more flexibility than the existing distributions in terms of the form of the hazard rate function. (iv) The proposed model is capable of fitting different kinds of data such as medical data and engineering data, as well as actuarial data which gives it a very interesting usage in many fields of sciences.

The following constitutes the presentation of this article: We provide the suggested distribution WBHD in Portion 2, along with its PDF and CDF functions. The graphical plots of the PDF and HRF are also presented in this section of the paper. In section 3, we establish several statistical features that are relevant to the WBHD. Eleven traditional approaches to estimating were discussed in Section 4. Also, in Section 4, the simulation research along with its numerical findings were carried out. Risk measures of our proposed model were discussed mathematically in Section 5. In Section 6, we are now going to do the real data analysis. Section 7 of this study piece is where the concluding observations are presented.

## 2 Formulation of the WBHD

In this section we define the formulation of the proposed model. Using the cumulative distribution function (CDF) of the WD-G [20], so we can define the CDF of the two-parameter WBHD as follows

$$\begin{array}{c} F\left(x\right)=\frac{\text{log}({\left(1-\frac{e^{-\alpha x}}{x+1}\right)}^{a}+1)}{\text{log}(2)}, \end{array}$$ (1)

and its probability density function (PDF) is defined as follows

$$\begin{array}{c} f\left(x\right)=\frac{a\left(\alpha +\alpha x+1\right){\left(1-\frac{e^{\alpha (-x)}}{x+1}\right)}^{a}}{\left(x+1\right)\text{log}\left(2\right)(\left(x+1\right)e^{\alpha x}-1)({\left(1-\frac{e^{\alpha (-x)}}{x+1}\right)}^{a}+1)}. \end{array}$$ (2)

The hazard rate function (HRF) of WBHD is defined as follows

$$\begin{array}{c} h\left(x\right)=\frac{a\left(\alpha +\alpha x+1\right){\left(1-\frac{e^{\alpha \left(-x\right)}}{x+1}\right)}^{a}}{\left(x+1\right)\left(\left(x+1\right)e^{\alpha x}-1\right)\left({\left(1-\frac{e^{\alpha \left(-x\right)}}{x+1}\right)}^{a}+1\right)\left(\text{log}\left(2\right)-\text{log}\left({\left(1-\frac{e^{\alpha \left(-x\right)}}{x+1}\right)}^{a}+1\right)\right)}. \end{array}$$ (3)

**Fig 1:**
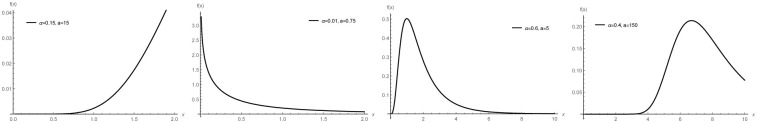
Plots of the PDFs of the WBHD.

**Fig 2:**
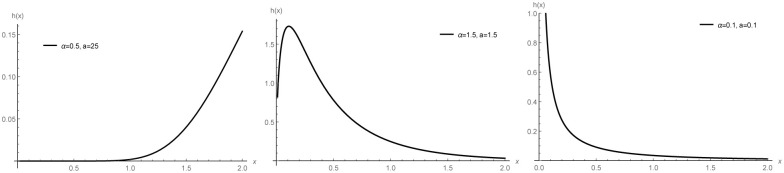
Plots of the HRFs of the WBHD.

Now we will graph all possible shapes of the PDF of the WBHD and HRF of the WBHD. In Fig 1, we provided three different possible shapes for the PDF of the WBHD: an increasing function, a decreasing function, and a unimodal function. Also, we provided the possible shapes of the HRF of the WBHD in Fig 2.

## 3 Statistical properties

Defining the mathematical properties of the proposed model is very essential and important to study the behaviour of the model and making computation easy, also generating data from the proposed distribution depends on the quantile function. This section contains a mathematical discussion of the statistical properties of the WBHD.

### 3.1 Quantile function

In order to calculate the quantile function (QF) of the WBHD, you must determine the inverse function of the CDF (1). This may be done as the following

$$\begin{array}{c} Q\left(p\right)=\frac{W\left(-\frac{e^{\alpha }\alpha }{{\left(2^{p}-1\right)}^{1/a}-1}\right)-\alpha }{\alpha },\;. \end{array}$$ (4)

where 0 < *p* < 1 and *W*(⋅) is Lambert function. It used to find the WBHD quarterlies, and to have randomly generated data sets by the following relation

$$\begin{array}{c} x_{i}=\frac{W\left(-\frac{e^{\alpha }\alpha }{{\left(2^{p_{i}}-1\right)}^{1/a}-1}\right)-\alpha }{\alpha },\;i=1,2,\ldots ,n. \end{array}$$

### 3.2 Linear representation

The CDF 1 and the PDF 2 of the proposed model can be linearly represented by using the following expansion $\text{log}\left(1+x\right)=\sum_{j=1}^{\infty }{\left(-1\right)}^{j+1}\frac{x^{j}}{j}$ as follows

$$\begin{array}{cc} F\left(x\right) & =\sum_{j=1}^{\infty }\Delta_{j}G_{j}\left(x\right),\\ f\left(x\right) & =\sum_{j=1}^{\infty }\Delta_{j}g_{j}\left(x\right), \end{array}$$

where $\Delta_{j}=\frac{{\left(-1\right)}^{j+1}}{j\text{log}\left(2\right)}$ and $G_{j}\left(x\right)={\left(1-\frac{e^{-\alpha x}}{x+1}\right)}^{aj}$ follows the exponentiated Burr-Hatke distribution (ExBHD).

### 3.3 Moments

The *q*th moments of the WBHD has the form

$$\begin{array}{cc} \mu_{q}^{\prime } & =\int_{0}^{\infty }x^{q}f\left(x\right)dx=\sum_{j=1}^{\infty }\Delta_{j}\int_{0}^{\infty }x^{q}g_{j}\left(x\right)dx\\ & =\sum_{j=1}^{\infty }\sum_{i,k=0}^{\infty }\Delta_{j}aj\;h\left(aj,i,k\right)\frac{\left(q+k+1\right)\left(i+1\right)\left(\alpha +1\right)}{{\left(j+1\right)}^{q+k+2}\alpha^{q+k+1}}\Gamma \left(q+k+1\right), \end{array}$$

where h(aj,i,k)=(-1)i+kΓ(aj)j!Γ(aj-i)(i+k+1k). Setting *q* = 1, 2, 3, and 4, respectively, we obtain the first four moments about the origin of the WBHD.

The nth central moment of X, say *μ*_*n*_, follows as

μn=E(x-μ)n=∑k=0∞(-1)k(nk)μ1′kμn-k′.

The cumulants (*k*_*n*_) of X can be obtained as the following

kn=μn′-∑k=0n-1(n-1k-1)krμn-r′.

### 3.4 Incomplete moments

The *d*th incomplete moment of WBHD is calculated as follows

$$\begin{array}{cc} T_{d}\left(t\right) & =\int_{0}^{t}x^{d}f\left(x\right)dx=\sum_{j=1}^{\infty }\Delta_{j}\int_{0}^{t}x^{d}f\left(x\right)dx\\ & =\sum_{j=1}^{\infty }\sum_{i,k,m=0}^{\infty }\Delta_{j}aj\;h\left(aj,i,k\right)[\frac{\alpha x^{d+k+m+2}}{d+k+m+2}+\frac{\left(\alpha +1\right)x^{d+k+m+1}}{d+k+m+1}]\frac{{\left[-\alpha \left(i+1\right)\right]}^{m}}{m!}. \end{array}$$

Many fields in our life may find great use for Lorenz curve which can be obtained by incomplete moments, $L\left(p\right)=\frac{T_{1}\left(x_{p}\right)}{\mu }$, *x*_*p*_ is the quantile function. One other use for the first incomplete moment is to calculate both the mean residual life and the mean waiting time, both of which are calculated using by *m*_1_(*t*) = [1 − *T*_1_(*t*)]/*S*(*t*) − *t* and *M*_1_(*t*) = *t* − *T*_1_(*t*)/*F*(*t*), respectively.

### 3.5 Order statistics

The PDF and CDF of the *i*th order statistic for the WBHD are

fi:n(x)=n!(i-1)!(n-i)![F(x)]i-1[1-F(x)]n-if(x)=an!log-i(2)(α+αx+1)(1-eα(-x)x+1)alogi-1((1-eα(-x)x+1)a+1)(1-log((1-eα(-x)x+1)a+1)log(2))n-i(x+1)(i-1)!((x+1)eαx-1)(n-i)!((1-eα(-x)x+1)a+1),Fi:n(x)=∑r=in(rn)(F(x))r(1-F(x))n-r=log-i(2)(ni)logi((1-eα(-x)x+1)a+1)(1-log((1-eα(-x)x+1)a+1)log(2))n-iY,

where $Y={\;}_{2}F_{1}\left(1,i-n;i+1;-\frac{\text{log}\left({\left(1-\frac{e^{-x\alpha }}{x+1}\right)}^{a}+1\right)}{\text{log}\left(2\right)-\text{log}\left({\left(1-\frac{e^{-x\alpha }}{x+1}\right)}^{a}+1\right)}\right)$ is a hyper geometric function.

## 4 Methods of estimation

This section discusses eleven techniques for estimating the WBHD’s parameters, *θ* = (*a*, *α*)^⊤^, and compares them using Monte Carlo simulations. To determine the estimates of *θ* in the following approaches, the **AdequacyModel** package for the the R software offers a thorough and effective universal meta-heuristic optimization method for maximizing or minimizing an arbitrary objective function. Visit https://rdrr.io/cran/AdequacyModel/ for more information.

### 4.1 Classical methods of estimation

i With respect to the WBHD parameters, the maximum likelihood estimation (MLE) is calculated by maximizing the log-likelihood function, which is described as follows (*x*_1_, …, *x*_*n*_ is a random sample from WBHD)
  $$\begin{array}{cc} l\left(x_{i};\theta \right) & =a\sum_{i=1}^{n}\text{log}\left(1-\frac{e^{\alpha \left(-x_{i}\right)}}{x_{i}+1}\right)-\sum_{i=1}^{n}\text{log}\left({\left(1-\frac{e^{\alpha \left(-x_{i}\right)}}{x_{i}+1}\right)}^{a}+1\right)+\sum_{i=1}^{n}\text{log}\left(\alpha +\alpha x_{i}+1\right)\\ & -\sum_{i=1}^{n}\text{log}\left(\left(x_{i}+1\right)e^{\alpha x_{i}}-1\right)-\sum_{i=1}^{n}\text{log}\left(x_{i}+1\right)+n\text{log}\left(\frac{a}{\text{log}\left(2\right)}\right). \end{array}$$
ii The Anderson-Darling estimation (ADE) is used to calculate the WBHD estimated parameters by minimizing the following equation (*x*_(1)_ ≤ *x*_(2)_ ≤ … ≤ *x*_(*n*)_)
  $$\begin{array}{cc} A\left(x_{i};\theta \right) & =-n-\frac{1}{n}\sum_{i=1}^{n}\left(2i-1\right)\left[\text{log}F\left(x_{i}\right)+\text{log}S\left(x_{i}\right)\right]\\ & =-n-\frac{1}{n}\sum_{i=1}^{n}\left(2i-1\right)[\text{log}\left(\frac{\text{log}\left({\left(1-\frac{e^{-\alpha x_{i}}}{x_{i}+1}\right)}^{a}+1\right)}{\text{log}\left(2\right)}\right)+\text{log}\left(1-\frac{\text{log}\left({\left(1-\frac{e^{-\alpha x_{i}}}{x_{i}+1}\right)}^{a}+1\right)}{\text{log}\left(2\right)}\right)]. \end{array}$$
iii The right-tail Anderson-Darling estimation (RADE) is used to calculate the WBHD estimated parameters by minimizing the following equation (*x*_(1)_ ≤ *x*_(2)_ ≤ … ≤ *x*_(*n*)_)
  $$\begin{array}{cc} R\left(x_{i};\theta \right) & =\frac{n}{2}-2\sum_{i=1}^{n}F\left(x_{i}\right)-\frac{1}{n}\sum_{i=1}^{n}\left(2i-1\right)\text{log}S\left(x_{n+1-i}\right)\\ & =\frac{n}{2}-2\sum_{i=1}^{n}\frac{\text{log}\left({\left(1-\frac{e^{-\alpha x_{i}}}{x_{i}+1}\right)}^{a}+1\right)}{\text{log}\left(2\right)}-\frac{1}{n}\sum_{i=1}^{n}\left(2i-1\right)\text{log}\left(\frac{\text{log}\left({\left(1-\frac{e^{-\alpha x_{n+1-i}}}{x_{n+1-i}+1}\right)}^{a}+1\right)}{\text{log}\left(2\right)}\right). \end{array}$$
iv The left-tailed Anderson-Darling estimation (LTADE) is used to calculate the WBHD estimated parameters by minimizing the following equation (*x*_(1)_ ≤ *x*_(2)_ ≤ … ≤ *x*_(*n*)_)
  $$\begin{array}{cc} L\left(x_{i};\theta \right) & =-\frac{3}{2}n+2\sum_{i=1}^{n}F\left(x_{i}\right)-\frac{1}{n}\sum_{i=1}^{n}\left(2i-1\right)\text{log}F\left(x_{i}\right)\\ & =-\frac{3}{2}n+2\sum_{i=1}^{n}\frac{\text{log}\left({\left(1-\frac{e^{-\alpha x_{i}}}{x_{i}+1}\right)}^{a}+1\right)}{\text{log}\left(2\right)}-\frac{1}{n}\sum_{i=1}^{n}\left(2i-1\right)\text{log}\frac{\text{log}\left({\left(1-\frac{e^{-\alpha x_{i}}}{x_{i}+1}\right)}^{a}+1\right)}{\text{log}\left(2\right)}. \end{array}$$
v The Cramér-von Mises estimation (CVME) is used to calculate the WBHD estimated parameters by minimizing the following equation (*x*_(1)_ ≤ *x*_(2)_ ≤ … ≤ *x*_(*n*)_)
  $$\begin{array}{cc} C\left(x_{i};\theta \right) & =-\frac{1}{12n}+\sum_{i=1}^{n}{\left[F\left(x_{i}\right)-\frac{2i-1}{2n}\right]}^{2}=-\frac{1}{12n}+\sum_{i=1}^{n}{\left[\frac{\text{log}\left({\left(1-\frac{e^{-\alpha x_{i}}}{x_{i}+1}\right)}^{a}+1\right)}{\text{log}\left(2\right)}-\frac{2i-1}{2n}\right]}^{2}. \end{array}$$
vi The least-squares estimation (LSE) is used to calculate the WBHD estimated parameters by minimizing the following equation (*x*_(1)_ ≤ *x*_(2)_ ≤ … ≤ *x*_(*n*)_)
  $$\begin{array}{c} V\left(x_{i};\theta \right)=\sum_{i=1}^{n}{\left[F\left(x_{i}\right)-\frac{i}{n+1}\right]}^{2}=\sum_{i=1}^{n}{\left[\frac{\text{log}\left({\left(1-\frac{e^{-\alpha x_{i}}}{x_{i}+1}\right)}^{a}+1\right)}{\text{log}\left(2\right)}-\frac{i}{n+1}\right]}^{2}. \end{array}$$
vii The weighted least-squares estimation (WLSE) is used to calculate the WBHD estimated parameters by minimizing the following equation (*x*_(1)_ ≤ *x*_(2)_ ≤ … ≤ *x*_(*n*)_)
  $$\begin{array}{cc} W\left(x_{i};\theta \right) & =\sum_{i=1}^{n}\frac{{\left(n+1\right)}^{2}\left(n+2\right)}{i\left(n-i+1\right)}{\left[F\left(x_{i}\right)-\frac{i}{n+1}\right]}^{2}\\ & =\sum_{i=1}^{n}\frac{{\left(n+1\right)}^{2}\left(n+2\right)}{i\left(n-i+1\right)}{\left[\frac{\text{log}\left({\left(1-\frac{e^{-\alpha x_{i}}}{x_{i}+1}\right)}^{a}+1\right)}{\text{log}\left(2\right)}-\frac{i}{n+1}\right]}^{2}. \end{array}$$
viii The maximum product of spacing estimation (MPSE) is used to calculate the WBHD estimated parameters by maximizing the following equation (*x*_(1)_ ≤ *x*_(2)_ ≤ … ≤ *x*_(*n*)_)
  $$\begin{array}{c} M\left(x_{i};\theta \right)=\frac{1}{n+1}\sum_{i=1}^{n+1}{\text{log}\;\psi }_{i}\left(x_{i};\theta \right), \end{array}$$
  where
  $$\begin{array}{cc} \psi_{i}\left(\alpha ,\lambda ,\xi \right) & =F\left(x_{i}\right)-F\left(x_{i-1}\right)=\frac{\text{log}\left({\left(1-\frac{e^{-\alpha x_{i}}}{x_{i}+1}\right)}^{a}+1\right)}{\text{log}\left(2\right)}-\frac{\text{log}\left({\left(1-\frac{e^{-\alpha x_{i-1}}}{x_{i-1}+1}\right)}^{a}+1\right)}{\text{log}\left(2\right)}. \end{array}$$
ix The minimum spacing absolute distance estimation (MSADE) is used to calculate the WBHD estimated parameters by minimizing the following equation (*x*_(1)_ ≤ *x*_(2)_ ≤ … ≤ *x*_(*n*)_)
  $$\begin{array}{cc} M_{1}\left(x_{i};\theta \right) & =\sum_{i=1}^{n+1}|\psi_{i}-\frac{1}{n+1}|. \end{array}$$
x The minimum spacing absolute-log distance estimation (MSALDE) is used to calculate the WBHD estimated parameters by minimizing the following equation (*x*_(1)_ ≤ *x*_(2)_ ≤ … ≤ *x*_(*n*)_)
  $$\begin{array}{cc} M_{2}\left(x_{i};\theta \right) & =\sum_{i=1}^{n+1}|{\text{log}\;\psi }_{i}-\text{log}\frac{1}{n+1}|. \end{array}$$
xi The percentile estimation (PE) is used to calculate the WBHD estimated parameters by minimizing the following equation (*x*_(1)_ ≤ *x*_(2)_ ≤ … ≤ *x*_(*n*)_)
  $$\begin{array}{c} PE\left(x_{i};\theta \right)=\sum_{i=1}^{n}{\left[x_{i}-Q\left(p_{i}\right)\right]}^{2}=\sum_{i=1}^{n}{\left[x_{i}-\frac{W\left(-\frac{e^{\alpha }\alpha }{{\left(2^{p_{i}}-1\right)}^{1/a}-1}\right)-\alpha }{\alpha }\right]}^{2}. \end{array}$$

### 4.2 Monte Carlo simulations

Using the results of simulations, we investigate how well the estimate approaches the initial values of the WBHD parameters. We will use the sample sizes n = 25, 60, 100, 200, 300, and 500, as well as various parameter values. We create *N* = 1, 000 random samples from the WBHD, and then we use the software package R to compute the average absolute biases (ABBs), mean square errors (MSEs), and mean relative estimates (MREs).

We explore the efficiency of the aforementioned estimate methodologies for computing the WBHD parameters using the simulation data. We will use several parameter values and the sample sizes n = 25, 60, 100, 200, 300, and 500. Using the software package R, we generate *N* = 1, 000 random samples from the WBHD and calculate the average absolute biases (ABBs), $ABBs=\frac{1}{N}\sum_{i=1}^{N}\left|\hat{\theta_{i}}-\theta_{i}\right|$, mean square errors (MSEs), $MSEs=\frac{1}{N}\sum_{i=1}^{N}{\left(\hat{\theta_{i}}-\theta_{i}\right)}^{2}$, and mean relative estimations (MREs), $MREs=\frac{1}{N}\sum_{i=1}^{N}\left|\hat{\theta_{i}}-\theta_{i}\right|/\theta_{i}$.

We find that the MPSE and MLE techniques are the best ways for estimating randomly generated data sets from WBHD, followed by the ADE method. Tables 1–5 give the numerical results of our simulation, while Table 6 reports ranks of each estimated method.

**Table 1:** Simulation values of BIAS, MSE and MRE for (*α* = 0.75, *a* = 0.25).

n	Est.	Est. Par.	MLE	ADE	CVME	MPSE	LSE	PCE	RTADE	WLSE	LTADE	MSADE	MSALDE
	BIAS	$\hat{\alpha }$	0.774777^{6}^	0.796688^{7}^	1.239129^{10}^	0.589766^{4}^	0.921732^{8}^	0.575242^{2}^	0.771367^{5}^	1.012331^{9}^	1.681914^{11}^	0.527217^{1}^	0.585158^{3}^
		$\hat{a}$	0.04323^{2}^	0.043584^{4}^	0.054392^{10}^	0.038382^{1}^	0.0464^{5}^	0.110172^{11}^	0.052489^{9}^	0.046546^{6}^	0.046706^{7}^	0.047691^{8}^	0.043283^{3}^
	MSE	$\hat{\alpha }$	1.857611^{6}^	2.007115^{7}^	4.910688^{9}^	0.783974^{2}^	2.462369^{8}^	0.473798^{1}^	1.728337^{5}^	5.177401^{10}^	17.031421^{11}^	1.07435^{3}^	1.112857^{4}^
		$\hat{a}$	0.003423^{3}^	0.003514^{4}^	0.005792^{10}^	0.002342^{1}^	0.004138^{8}^	0.022125^{11}^	0.005378^{9}^	0.00404^{6}^	0.004103^{7}^	0.003982^{5}^	0.003018^{2}^
	MRE	$\hat{\alpha }$	1.033036^{6}^	1.062251^{7}^	1.652172^{10}^	0.786355^{4}^	1.228976^{8}^	0.76699^{2}^	1.02849^{5}^	1.349775^{9}^	2.242552^{11}^	0.702956^{1}^	0.780211^{3}^
		$\hat{a}$	0.172922^{2}^	0.174335^{4}^	0.21757^{10}^	0.153529^{1}^	0.1856^{5}^	0.44069^{11}^	0.209956^{9}^	0.186183^{6}^	0.186823^{7}^	0.190765^{8}^	0.173131^{3}^
	∑*Ranks*		25^{3}^	34^{5}^	60^{11}^	13^{1}^	43^{8}^	38^{6}^	42^{7}^	47^{9}^	50^{10}^	26^{4}^	18^{2}^
	BIAS	$\hat{\alpha }$	0.404122^{4}^	0.472518^{7}^	0.63033^{10}^	0.373487^{3}^	0.592335^{9}^	0.446524^{6}^	0.439698^{5}^	0.527187^{8}^	0.70094^{11}^	0.321462^{1}^	0.357929^{2}^
		$\hat{a}$	0.02703^{4}^	0.02562^{1}^	0.03038^{8}^	0.025847^{2}^	0.029039^{6}^	0.082331^{11}^	0.03167^{9}^	0.02812^{5}^	0.029074^{7}^	0.032672^{10}^	0.026966^{3}^
	MSE	$\hat{\alpha }$	0.351797^{5}^	0.447417^{6}^	0.88663^{10}^	0.254503^{3}^	0.760036^{9}^	0.306716^{4}^	0.463295^{7}^	0.713467^{8}^	1.126514^{11}^	0.244724^{1}^	0.244766^{2}^
		$\hat{a}$	0.001174^{4}^	0.001085^{2}^	0.001615^{8}^	0.001022^{1}^	0.001455^{7}^	0.011202^{11}^	0.001743^{10}^	0.001294^{5}^	0.001409^{6}^	0.001729^{9}^	0.001163^{3}^
	MRE	$\hat{\alpha }$	0.538829^{4}^	0.630024^{7}^	0.84044^{10}^	0.497983^{3}^	0.789781^{9}^	0.595366^{6}^	0.586264^{5}^	0.702915^{8}^	0.934586^{11}^	0.428616^{1}^	0.477239^{2}^
		$\hat{a}$	0.108118^{4}^	0.10248^{1}^	0.12152^{8}^	0.103388^{2}^	0.116155^{6}^	0.329322^{11}^	0.12668^{9}^	0.112479^{5}^	0.116294^{7}^	0.130689^{10}^	0.107864^{3}^
	∑*Ranks*		25^{4}^	24^{3}^	54^{11}^	14^{1}^	46^{8}^	49^{9}^	45^{7}^	39^{6}^	53^{10}^	32^{5}^	15^{2}^
	BIAS	$\hat{\alpha }$	0.277807^{3}^	0.308074^{5}^	0.433779^{10}^	0.263195^{2}^	0.425532^{9}^	0.365708^{8}^	0.329597^{6}^	0.361046^{7}^	0.520825^{11}^	0.246823^{1}^	0.289087^{4}^
		$\hat{a}$	0.019402^{2}^	0.020149^{3}^	0.022752^{7}^	0.018907^{1}^	0.02368^{9}^	0.072277^{11}^	0.023366^{8}^	0.021685^{4}^	0.022677^{6}^	0.024266^{10}^	0.022147^{5}^
	MSE	$\hat{\alpha }$	0.150966^{4}^	0.173358^{5}^	0.374387^{10}^	0.1104^{1}^	0.323512^{9}^	0.213844^{7}^	0.193063^{6}^	0.248867^{8}^	0.609919^{11}^	0.117603^{2}^	0.139287^{3}^
		$\hat{a}$	0.000615^{2}^	0.000681^{3}^	0.000816^{6}^	0.000564^{1}^	0.000896^{9}^	0.008395^{11}^	0.000865^{7}^	0.000751^{4}^	0.000868^{8}^	0.00091^{10}^	0.000781^{5}^
	MRE	$\hat{\alpha }$	0.370409^{3}^	0.410765^{5}^	0.578372^{10}^	0.350927^{2}^	0.567376^{9}^	0.48761^{8}^	0.439462^{6}^	0.481395^{7}^	0.694433^{11}^	0.329097^{1}^	0.38545^{4}^
		$\hat{a}$	0.077607^{2}^	0.080597^{3}^	0.091008^{7}^	0.075628^{1}^	0.094721^{9}^	0.289109^{11}^	0.093463^{8}^	0.086741^{4}^	0.090709^{6}^	0.097064^{10}^	0.08859^{5}^
	∑*Ranks*		16^{2}^	24^{3}^	50^{8}^	8^{1}^	54^{10}^	56^{11}^	41^{7}^	34^{5.5}^	53^{9}^	34^{5.5}^	26^{4}^
	BIAS	$\hat{\alpha }$	0.188306^{2}^	0.220352^{6}^	0.286808^{9}^	0.18692^{1}^	0.289854^{10}^	0.267068^{8}^	0.206482^{5}^	0.253405^{7}^	0.350714^{11}^	0.200719^{4}^	0.200069^{3}^
		$\hat{a}$	0.0135^{2}^	0.014474^{3}^	0.015855^{6}^	0.013461^{1}^	0.016055^{8}^	0.053393^{11}^	0.016047^{7}^	0.015109^{4}^	0.016515^{9}^	0.017396^{10}^	0.015767^{5}^
	MSE	$\hat{\alpha }$	0.065728^{2}^	0.082027^{6}^	0.144405^{9}^	0.05556^{1}^	0.152483^{10}^	0.110201^{7}^	0.072175^{4}^	0.113992^{8}^	0.219538^{11}^	0.074191^{5}^	0.069452^{3}^
		$\hat{a}$	0.000297^{2}^	0.000339^{3}^	0.000402^{6}^	0.000292^{1}^	0.000406^{7}^	0.004413^{11}^	0.000422^{8}^	0.000365^{4}^	0.000425^{9}^	0.000484^{10}^	4*e* − 04^{5}^
	MRE	$\hat{\alpha }$	0.251075^{2}^	0.293802^{6}^	0.38241^{9}^	0.249226^{1}^	0.386471^{10}^	0.356091^{8}^	0.27531^{5}^	0.337873^{7}^	0.467619^{11}^	0.267625^{4}^	0.266758^{3}^
		$\hat{a}$	0.054001^{2}^	0.057894^{3}^	0.063419^{6}^	0.053844^{1}^	0.06422^{8}^	0.213573^{11}^	0.064187^{7}^	0.060435^{4}^	0.066061^{9}^	0.069585^{10}^	0.063068^{5}^
	∑*Ranks*		12^{2}^	27^{3}^	44^{8}^	6^{1}^	52^{9}^	55^{10}^	35^{6}^	34^{5}^	59^{11}^	42^{7}^	30^{4}^
	BIAS	$\hat{\alpha }$	0.150814^{2}^	0.191832^{7}^	0.248156^{10}^	0.141545^{1}^	0.24015^{9}^	0.226807^{8}^	0.172931^{5}^	0.190106^{6}^	0.286747^{11}^	0.159098^{4}^	0.152685^{3}^
		$\hat{a}$	0.011054^{2}^	0.011188^{3}^	0.013489^{9}^	0.01052^{1}^	0.012391^{5}^	0.047129^{11}^	0.013454^{8}^	0.011658^{4}^	0.012581^{6}^	0.014302^{10}^	0.01263^{7}^
	MSE	$\hat{\alpha }$	0.041795^{2}^	0.061013^{7}^	0.10763^{10}^	0.034073^{1}^	0.09253^{9}^	0.081918^{8}^	0.049701^{5}^	0.060745^{6}^	0.141928^{11}^	0.046232^{4}^	0.042113^{3}^
		$\hat{a}$	0.000196^{2}^	0.000213^{3}^	0.000291^{9}^	0.000184^{1}^	0.00025^{5}^	0.003571^{11}^	0.000288^{8}^	0.000224^{4}^	0.000252^{6}^	0.000325^{10}^	0.000261^{7}^
	MRE	$\hat{\alpha }$	0.201086^{2}^	0.255776^{7}^	0.330875^{10}^	0.188726^{1}^	0.320199^{9}^	0.302409^{8}^	0.230575^{5}^	0.253474^{6}^	0.382329^{11}^	0.21213^{4}^	0.20358^{3}^
		$\hat{a}$	0.044215^{2}^	0.044753^{3}^	0.053958^{9}^	0.042082^{1}^	0.049565^{5}^	0.188517^{11}^	0.053815^{8}^	0.046633^{4}^	0.050326^{6}^	0.05721^{10}^	0.050521^{7}^
	∑*Ranks*		12^{2}^	30^{4}^	57^{10.5}^	6^{1}^	42^{7.5}^	57^{10.5}^	39^{6}^	30^{4}^	51^{9}^	42^{7.5}^	30^{4}^
	BIAS	$\hat{\alpha }$	0.111925^{2}^	0.135004^{5}^	0.182087^{9}^	0.101345^{1}^	0.182986^{10}^	0.179779^{8}^	0.138011^{6}^	0.146853^{7}^	0.221565^{11}^	0.124982^{4}^	0.117584^{3}^
		$\hat{a}$	0.008145^{2}^	0.008611^{3}^	0.009968^{7}^	0.00811^{1}^	0.01047^{9}^	0.039038^{11}^	0.010119^{8}^	0.009182^{4}^	0.009376^{5}^	0.011249^{10}^	0.009591^{6}^
	MSE	$\hat{\alpha }$	0.022929^{2}^	0.031216^{5}^	0.056871^{10}^	0.017784^{1}^	0.053027^{9}^	0.050137^{8}^	0.031508^{6}^	0.03675^{7}^	0.079326^{11}^	0.029532^{4}^	0.024949^{3}^
		$\hat{a}$	0.000116^{2}^	0.00012^{3}^	0.000156^{7}^	0.00011^{1}^	0.000171^{9}^	0.002425^{11}^	0.000164^{8}^	0.000135^{4}^	0.000136^{5}^	0.000196^{10}^	0.00015^{6}^
	MRE	$\hat{\alpha }$	0.149233^{2}^	0.180005^{5}^	0.242782^{9}^	0.135127^{1}^	0.243982^{10}^	0.239706^{8}^	0.184015^{6}^	0.195805^{7}^	0.29542^{11}^	0.166642^{4}^	0.156779^{3}^
		$\hat{a}$	0.032578^{2}^	0.034445^{3}^	0.039873^{7}^	0.032441^{1}^	0.041881^{9}^	0.156151^{11}^	0.040477^{8}^	0.036729^{4}^	0.037505^{5}^	0.044997^{10}^	0.038365^{6}^
	∑*Ranks*		12^{2}^	24^{3}^	49^{9}^	6^{1}^	56^{10}^	57^{11}^	42^{6.5}^	33^{5}^	48^{8}^	42^{6.5}^	27^{4}^

**Table 2:** Simulation values of BIAS, MSE and MRE for (*α* = 1.5, *a* = 0.75).

n	Est.	Est. Par.	MLE	ADE	CVME	MPSE	LSE	PCE	RTADE	WLSE	LTADE	MSADE	MSALDE
	BIAS	$\hat{\alpha }$	0.640093^{4}^	0.641683^{5}^	0.861842^{10}^	0.600496^{2}^	0.74178^{9}^	0.728272^{8}^	0.672678^{7}^	0.669569^{6}^	0.887624^{11}^	0.587423^{1}^	0.605147^{3}^
		$\hat{a}$	0.147271^{2}^	0.152018^{3}^	0.189437^{9}^	0.145566^{1}^	0.164403^{7}^	0.279716^{11}^	0.193899^{10}^	0.160445^{5}^	0.163597^{6}^	0.169896^{8}^	0.153276^{4}^
	MSE	$\hat{\alpha }$	0.842388^{6}^	0.820489^{4}^	1.625904^{10}^	0.574683^{1}^	1.095676^{9}^	0.831712^{5}^	0.97254^{8}^	0.893759^{7}^	1.699768^{11}^	0.704929^{3}^	0.603267^{2}^
		$\hat{a}$	0.04149^{3}^	0.043148^{4}^	0.074224^{9}^	0.034739^{1}^	0.053284^{8}^	0.144685^{11}^	0.085259^{10}^	0.050118^{5}^	0.052383^{7}^	0.050845^{6}^	0.038839^{2}^
	MRE	$\hat{\alpha }$	0.426729^{4}^	0.427789^{5}^	0.574562^{10}^	0.40033^{2}^	0.49452^{9}^	0.485515^{8}^	0.448452^{7}^	0.446379^{6}^	0.591749^{11}^	0.391615^{1}^	0.403432^{3}^
		$\hat{a}$	0.196361^{2}^	0.20269^{3}^	0.252582^{9}^	0.194088^{1}^	0.219204^{7}^	0.372955^{11}^	0.258532^{10}^	0.213927^{5}^	0.218129^{6}^	0.226528^{8}^	0.204368^{4}^
	∑*Ranks*		21^{3}^	24^{4}^	57^{11}^	8^{1}^	49^{7}^	54^{10}^	52^{8.5}^	34^{6}^	52^{8.5}^	27^{5}^	18^{2}^
	BIAS	$\hat{\alpha }$	0.374034^{1}^	0.412634^{7}^	0.479236^{9}^	0.376106^{2}^	0.451375^{8}^	0.519836^{10}^	0.395609^{3}^	0.410144^{6}^	0.531449^{11}^	0.399458^{4}^	0.407639^{5}^
		$\hat{a}$	0.092891^{3}^	0.095353^{4}^	0.106606^{8}^	0.087626^{1}^	0.100199^{6}^	0.196645^{11}^	0.107187^{10}^	0.090314^{2}^	0.103392^{7}^	0.106779^{9}^	0.098734^{5}^
	MSE	$\hat{\alpha }$	0.242031^{2}^	0.295418^{5}^	0.39091^{9}^	0.219732^{1}^	0.347119^{8}^	0.405006^{10}^	0.282238^{4}^	0.296817^{6}^	0.519362^{11}^	0.301336^{7}^	0.259648^{3}^
		$\hat{a}$	0.015075^{4}^	0.015603^{5}^	0.020563^{10}^	0.012058^{1}^	0.017317^{6}^	0.05935^{11}^	0.020367^{9}^	0.014082^{2}^	0.018523^{8}^	0.018031^{7}^	0.014637^{3}^
	MRE	$\hat{\alpha }$	0.249356^{1}^	0.275089^{7}^	0.319491^{9}^	0.250738^{2}^	0.300917^{8}^	0.346557^{10}^	0.263739^{3}^	0.273429^{6}^	0.354299^{11}^	0.266305^{4}^	0.271759^{5}^
		$\hat{a}$	0.123855^{3}^	0.127137^{4}^	0.142141^{8}^	0.116835^{1}^	0.133599^{6}^	0.262193^{11}^	0.142916^{10}^	0.120418^{2}^	0.137856^{7}^	0.142372^{9}^	0.131645^{5}^
	∑*Ranks*		14^{2}^	32^{5}^	53^{9}^	8^{1}^	42^{8}^	63^{11}^	39^{6}^	24^{3}^	55^{10}^	40^{7}^	26^{4}^
	BIAS	$\hat{\alpha }$	0.268268^{1}^	0.315205^{5}^	0.362121^{9}^	0.293166^{2}^	0.347421^{8}^	0.381137^{10}^	0.312587^{4}^	0.317269^{6}^	0.386594^{11}^	0.320422^{7}^	0.307712^{3}^
		$\hat{a}$	0.066305^{2}^	0.071412^{3}^	0.078868^{8}^	0.065458^{1}^	0.078867^{7}^	0.155181^{11}^	0.083255^{9}^	0.073039^{4}^	0.075689^{5}^	0.084958^{10}^	0.0774^{6}^
	MSE	$\hat{\alpha }$	0.121061^{1}^	0.167018^{5}^	0.222633^{9}^	0.135428^{2}^	0.202487^{8}^	0.223999^{10}^	0.161002^{4}^	0.169713^{6}^	0.2481^{11}^	0.186481^{7}^	0.145122^{3}^
		$\hat{a}$	0.007001^{2}^	0.008092^{3}^	0.010484^{8}^	0.006793^{1}^	0.009895^{7}^	0.038007^{11}^	0.011649^{10}^	0.009109^{4}^	0.009491^{6}^	0.011535^{9}^	0.009471^{5}^
	MRE	$\hat{\alpha }$	0.178846^{1}^	0.210137^{5}^	0.241414^{9}^	0.195444^{2}^	0.231614^{8}^	0.254091^{10}^	0.208391^{4}^	0.211512^{6}^	0.257729^{11}^	0.213615^{7}^	0.205141^{3}^
		$\hat{a}$	0.088406^{2}^	0.095216^{3}^	0.105157^{7.5}^	0.087277^{1}^	0.105157^{7.5}^	0.206908^{11}^	0.111007^{9}^	0.097385^{4}^	0.100919^{5}^	0.113278^{10}^	0.1032^{6}^
	∑*Ranks*		9^{1.5}^	24^{3}^	50.5^{10}^	9^{1.5}^	45.5^{7}^	63^{11}^	40^{6}^	30^{5}^	49^{8}^	50^{9}^	26^{4}^
	BIAS	$\hat{\alpha }$	0.202301^{1}^	0.203177^{2}^	0.250781^{9}^	0.206198^{3}^	0.242439^{8}^	0.293548^{11}^	0.218143^{5}^	0.215205^{4}^	0.268887^{10}^	0.225232^{6}^	0.231009^{7}^
		$\hat{a}$	0.048285^{2}^	0.04787^{1}^	0.056865^{7}^	0.050426^{4}^	0.05499^{6}^	0.126695^{11}^	0.058901^{9}^	0.04928^{3}^	0.057111^{8}^	0.059489^{10}^	0.053622^{5}^
	MSE	$\hat{\alpha }$	0.06751^{3}^	0.066173^{2}^	0.103566^{9}^	0.064706^{1}^	0.094217^{8}^	0.13394^{11}^	0.074201^{4}^	0.077846^{5}^	0.115265^{10}^	0.086454^{7}^	0.082228^{6}^
		$\hat{a}$	0.003783^{2}^	0.003639^{1}^	0.005366^{8}^	0.003905^{3}^	0.004844^{6}^	0.024355^{11}^	0.005446^{9}^	0.003977^{4}^	0.005088^{7}^	0.005671^{10}^	0.004583^{5}^
	MRE	$\hat{\alpha }$	0.134867^{1}^	0.135451^{2}^	0.167188^{9}^	0.137466^{3}^	0.161626^{8}^	0.195698^{11}^	0.145429^{5}^	0.14347^{4}^	0.179258^{10}^	0.150155^{6}^	0.154006^{7}^
		$\hat{a}$	0.064381^{2}^	0.063826^{1}^	0.075821^{7}^	0.067234^{4}^	0.07332^{6}^	0.168926^{11}^	0.078535^{9}^	0.065707^{3}^	0.076147^{8}^	0.079318^{10}^	0.071496^{5}^
	∑*Ranks*		11^{2}^	9^{1}^	49^{8.5}^	18^{3}^	42^{7}^	66^{11}^	41^{6}^	23^{4}^	53^{10}^	49^{8.5}^	35^{5}^
	BIAS	$\hat{\alpha }$	0.160385^{1}^	0.188599^{7}^	0.196387^{8}^	0.160637^{2}^	0.200755^{9}^	0.235352^{11}^	0.178952^{4}^	0.173333^{3}^	0.229417^{10}^	0.187717^{6}^	0.186466^{5}^
		$\hat{a}$	0.03772^{1}^	0.041978^{4}^	0.04563^{8}^	0.037962^{2}^	0.045191^{6}^	0.1026^{11}^	0.047146^{9}^	0.041294^{3}^	0.04368^{5}^	0.048012^{10}^	0.045406^{7}^
	MSE	$\hat{\alpha }$	0.041418^{2}^	0.057649^{7}^	0.061722^{8}^	0.040821^{1}^	0.065666^{9}^	0.086359^{11}^	0.050206^{4}^	0.049083^{3}^	0.085633^{10}^	0.056992^{6}^	0.055555^{5}^
		$\hat{a}$	0.002262^{2}^	0.002711^{3}^	0.003259^{7}^	0.002252^{1}^	0.003315^{8}^	0.016787^{11}^	0.003571^{9}^	0.002825^{4}^	0.003062^{5}^	0.003624^{10}^	0.003201^{6}^
	MRE	$\hat{\alpha }$	0.106923^{1}^	0.125733^{7}^	0.130925^{8}^	0.107092^{2}^	0.133837^{9}^	0.156901^{11}^	0.119302^{4}^	0.115555^{3}^	0.152945^{10}^	0.125145^{6}^	0.124311^{5}^
		$\hat{a}$	0.050293^{1}^	0.055971^{4}^	0.06084^{8}^	0.050615^{2}^	0.060254^{6}^	0.136799^{11}^	0.062862^{9}^	0.055058^{3}^	0.05824^{5}^	0.064016^{10}^	0.060542^{7}^
	∑*Ranks*		8^{1}^	32^{4}^	47^{8.5}^	10^{2}^	47^{8.5}^	66^{11}^	39^{6}^	19^{3}^	45^{7}^	48^{10}^	35^{5}^
	BIAS	$\hat{\alpha }$	0.121213^{1}^	0.136753^{4}^	0.150165^{9}^	0.125928^{2}^	0.147264^{7}^	0.193262^{11}^	0.138169^{5}^	0.13384^{3}^	0.169791^{10}^	0.149766^{8}^	0.142123^{6}^
		$\hat{a}$	0.032035^{4}^	0.030966^{2}^	0.035028^{8}^	0.030654^{1}^	0.034669^{7}^	0.084642^{11}^	0.035677^{9}^	0.030992^{3}^	0.032909^{5}^	0.03822^{10}^	0.034518^{6}^
	MSE	$\hat{\alpha }$	0.024163^{1}^	0.031506^{5}^	0.036434^{8}^	0.02621^{2}^	0.03427^{7}^	0.0576^{11}^	0.029642^{4}^	0.02839^{3}^	0.046849^{10}^	0.03695^{9}^	0.03222^{6}^
		$\hat{a}$	0.001629^{4}^	0.001531^{3}^	0.001915^{8}^	0.001446^{1}^	0.001902^{7}^	0.011205^{11}^	0.002051^{9}^	0.001508^{2}^	0.00172^{5}^	0.002266^{10}^	0.001887^{6}^
	MRE	$\hat{\alpha }$	0.080809^{1}^	0.091169^{4}^	0.10011^{9}^	0.083952^{2}^	0.098176^{7}^	0.128841^{11}^	0.092113^{5}^	0.089226^{3}^	0.113194^{10}^	0.099844^{8}^	0.094749^{6}^
		$\hat{a}$	0.042713^{4}^	0.041289^{2}^	0.046704^{8}^	0.040872^{1}^	0.046226^{7}^	0.112856^{11}^	0.047569^{9}^	0.041323^{3}^	0.043878^{5}^	0.05096^{10}^	0.046024^{6}^
	∑*Ranks*		15^{2}^	20^{4}^	50^{9}^	9^{1}^	42^{7}^	66^{11}^	41^{6}^	17^{3}^	45^{8}^	55^{10}^	36^{5}^

**Table 3:** Simulation values of BIAS, MSE and MRE for (*α* = 2.5, *a* = 1.5).

n	Est.	Est. Par.	MLE	ADE	CVME	MPSE	LSE	PCE	RTADE	WLSE	LTADE	MSADE	MSALDE
	BIAS	$\hat{\alpha }$	0.744687^{4}^	0.709672^{2}^	0.922934^{11}^	0.71092^{3}^	0.784401^{6}^	0.905243^{9}^	0.798215^{7}^	0.802425^{8}^	0.912696^{10}^	0.698705^{1}^	0.762354^{5}^
		$\hat{a}$	0.363081^{5}^	0.340471^{2}^	0.465367^{9}^	0.323963^{1}^	0.40762^{8}^	0.535425^{11}^	0.470912^{10}^	0.385827^{6}^	0.391987^{7}^	0.348983^{3}^	0.359483^{4}^
	MSE	$\hat{\alpha }$	1.02954^{5}^	0.886857^{3}^	1.705096^{11}^	0.750673^{1}^	1.164325^{7}^	1.272031^{9}^	1.199029^{8}^	1.141993^{6}^	1.621641^{10}^	0.885091^{2}^	0.91387^{4}^
		$\hat{a}$	0.288154^{5}^	0.229427^{4}^	0.584048^{10}^	0.166189^{1}^	0.433453^{8}^	0.494979^{9}^	0.625468^{11}^	0.306594^{6}^	0.375088^{7}^	0.223759^{3}^	0.219587^{2}^
	MRE	$\hat{\alpha }$	0.297875^{4}^	0.283869^{2}^	0.369174^{11}^	0.284368^{3}^	0.313761^{6}^	0.362097^{9}^	0.319286^{7}^	0.32097^{8}^	0.365078^{10}^	0.279482^{1}^	0.304942^{5}^
		$\hat{a}$	0.242054^{5}^	0.226981^{2}^	0.310245^{9}^	0.215975^{1}^	0.271747^{8}^	0.35695^{11}^	0.313941^{10}^	0.257218^{6}^	0.261325^{7}^	0.232656^{3}^	0.239655^{4}^
	∑*Ranks*		28^{5}^	15^{3}^	61^{11}^	10^{1}^	43^{7}^	58^{10}^	53^{9}^	40^{6}^	51^{8}^	13^{2}^	24^{4}^
	BIAS	$\hat{\alpha }$	0.451791^{1}^	0.459675^{3}^	0.533931^{9}^	0.454123^{2}^	0.501755^{8}^	0.591369^{11}^	0.479994^{5}^	0.477756^{4}^	0.581351^{10}^	0.492721^{7}^	0.481653^{6}^
		$\hat{a}$	0.221736^{5}^	0.21807^{3}^	0.255234^{9}^	0.203191^{1}^	0.236891^{6}^	0.357176^{11}^	0.267836^{10}^	0.213281^{2}^	0.238999^{7}^	0.243438^{8}^	0.220103^{4}^
	MSE	$\hat{\alpha }$	0.334086^{2}^	0.345062^{3}^	0.475017^{9}^	0.30858^{1}^	0.416448^{7}^	0.535295^{10}^	0.360495^{5}^	0.365701^{6}^	0.57755^{11}^	0.418516^{8}^	0.358509^{4}^
		$\hat{a}$	0.089596^{5}^	0.077336^{3}^	0.116963^{9}^	0.064186^{1}^	0.098108^{6}^	0.200666^{11}^	0.125962^{10}^	0.079666^{4}^	0.100782^{7}^	0.103288^{8}^	0.074388^{2}^
	MRE	$\hat{\alpha }$	0.180716^{1}^	0.18387^{3}^	0.213572^{9}^	0.181649^{2}^	0.200702^{8}^	0.236548^{11}^	0.191997^{5}^	0.191102^{4}^	0.23254^{10}^	0.197088^{7}^	0.192661^{6}^
		$\hat{a}$	0.147824^{5}^	0.14538^{3}^	0.170156^{9}^	0.13546^{1}^	0.157927^{6}^	0.238117^{11}^	0.178557^{10}^	0.142188^{2}^	0.159333^{7}^	0.162292^{8}^	0.146736^{4}^
	∑*Ranks*		19^{3}^	18^{2}^	54^{10}^	8^{1}^	41^{6}^	65^{11}^	45^{7}^	22^{4}^	52^{9}^	46^{8}^	26^{5}^
	BIAS	$\hat{\alpha }$	0.333132^{2}^	0.358823^{4}^	0.39802^{9}^	0.327761^{1}^	0.396629^{7}^	0.463167^{11}^	0.374139^{5}^	0.353366^{3}^	0.397423^{8}^	0.417475^{10}^	0.381586^{6}^
		$\hat{a}$	0.157752^{2}^	0.166399^{4}^	0.188226^{8}^	0.154898^{1}^	0.17926^{6}^	0.289818^{11}^	0.201402^{10}^	0.174037^{5}^	0.162923^{3}^	0.19618^{9}^	0.179613^{7}^
	MSE	$\hat{\alpha }$	0.187085^{2}^	0.209587^{4}^	0.27152^{9}^	0.164272^{1}^	0.256362^{8}^	0.33848^{11}^	0.235063^{6}^	0.202725^{3}^	0.255116^{7}^	0.287622^{10}^	0.227921^{5}^
		$\hat{a}$	0.041895^{2}^	0.046129^{4}^	0.061468^{8}^	0.036296^{1}^	0.053828^{7}^	0.129145^{11}^	0.07017^{10}^	0.050791^{6}^	0.044632^{3}^	0.064667^{9}^	0.049365^{5}^
	MRE	$\hat{\alpha }$	0.133253^{2}^	0.143529^{4}^	0.159208^{9}^	0.131104^{1}^	0.158652^{7}^	0.185267^{11}^	0.149655^{5}^	0.141346^{3}^	0.158969^{8}^	0.16699^{10}^	0.152635^{6}^
		$\hat{a}$	0.105168^{2}^	0.110932^{4}^	0.125484^{8}^	0.103265^{1}^	0.119506^{6}^	0.193212^{11}^	0.134268^{10}^	0.116025^{5}^	0.108615^{3}^	0.130787^{9}^	0.119742^{7}^
	∑*Ranks*		12^{2}^	24^{3}^	51^{9}^	6^{1}^	41^{7}^	66^{11}^	46^{8}^	25^{4}^	32^{5}^	57^{10}^	36^{6}^
	BIAS	$\hat{\alpha }$	0.232346^{1}^	0.242466^{3}^	0.278334^{7}^	0.242094^{2}^	0.278492^{8}^	0.350285^{11}^	0.25519^{5}^	0.255015^{4}^	0.299388^{10}^	0.281626^{9}^	0.272329^{6}^
		$\hat{a}$	0.10643^{1}^	0.115647^{3}^	0.130431^{7}^	0.11072^{2}^	0.131244^{8}^	0.230198^{11}^	0.135078^{9}^	0.116526^{4}^	0.127591^{6}^	0.137533^{10}^	0.124746^{5}^
	MSE	$\hat{\alpha }$	0.087269^{1}^	0.094521^{3}^	0.125151^{8}^	0.093291^{2}^	0.123508^{7}^	0.195419^{11}^	0.10378^{5}^	0.103215^{4}^	0.141751^{10}^	0.130255^{9}^	0.115004^{6}^
		$\hat{a}$	0.018787^{1}^	0.021252^{3}^	0.027582^{7}^	0.019097^{2}^	0.027986^{8}^	0.082737^{11}^	0.030851^{10}^	0.021614^{4}^	0.025823^{6}^	0.029524^{9}^	0.02369^{5}^
	MRE	$\hat{\alpha }$	0.092938^{1}^	0.096987^{3}^	0.111333^{7}^	0.096838^{2}^	0.111397^{8}^	0.140114^{11}^	0.102076^{5}^	0.102006^{4}^	0.119755^{10}^	0.11265^{9}^	0.108931^{6}^
		$\hat{a}$	0.070954^{1}^	0.077098^{3}^	0.086954^{7}^	0.073813^{2}^	0.087496^{8}^	0.153465^{11}^	0.090052^{9}^	0.077684^{4}^	0.085061^{6}^	0.091689^{10}^	0.083164^{5}^
	∑*Ranks*		6^{1}^	18^{3}^	43^{6.5}^	12^{2}^	47^{8}^	66^{11}^	43^{6.5}^	24^{4}^	48^{9}^	56^{10}^	33^{5}^
	BIAS	$\hat{\alpha }$	0.194446^{1}^	0.20337^{3}^	0.219672^{6}^	0.197492^{2}^	0.23779^{9}^	0.298271^{11}^	0.208349^{5}^	0.205849^{4}^	0.232504^{8}^	0.240895^{10}^	0.221937^{7}^
		$\hat{a}$	0.088973^{2}^	0.09587^{4}^	0.106323^{7}^	0.087394^{1}^	0.106716^{8}^	0.193928^{11}^	0.108973^{9}^	0.095473^{3}^	0.098068^{5}^	0.112094^{10}^	0.101894^{6}^
	MSE	$\hat{\alpha }$	0.06036^{1}^	0.06477^{3}^	0.078774^{7}^	0.060475^{2}^	0.08879^{9}^	0.136617^{11}^	0.069465^{5}^	0.066566^{4}^	0.088237^{8}^	0.096773^{10}^	0.076933^{6}^
		$\hat{a}$	0.012627^{2}^	0.014977^{4}^	0.018324^{7}^	0.011732^{1}^	0.018525^{8}^	0.057909^{11}^	0.019759^{9}^	0.014149^{3}^	0.015538^{5}^	0.020435^{10}^	0.016304^{6}^
	MRE	$\hat{\alpha }$	0.077778^{1}^	0.081348^{3}^	0.087869^{6}^	0.078997^{2}^	0.095116^{9}^	0.119308^{11}^	0.08334^{5}^	0.08234^{4}^	0.093002^{8}^	0.096358^{10}^	0.088775^{7}^
		$\hat{a}$	0.059315^{2}^	0.063913^{4}^	0.070882^{7}^	0.058263^{1}^	0.071144^{8}^	0.129285^{11}^	0.072649^{9}^	0.063649^{3}^	0.065379^{5}^	0.074729^{10}^	0.06793^{6}^
	∑*Ranks*		9^{1.5}^	21^{3.5}^	40^{7}^	9^{1.5}^	51^{9}^	66^{11}^	42^{8}^	21^{3.5}^	39^{6}^	60^{10}^	38^{5}^
	BIAS	$\hat{\alpha }$	0.142186^{1}^	0.150608^{3}^	0.178176^{8}^	0.147611^{2}^	0.169993^{7}^	0.224758^{11}^	0.159798^{5}^	0.154913^{4}^	0.186919^{10}^	0.185717^{9}^	0.1655^{6}^
		$\hat{a}$	0.069661^{1}^	0.072913^{3}^	0.084508^{8}^	0.071689^{2}^	0.080804^{7}^	0.1457^{11}^	0.085168^{9}^	0.072983^{4}^	0.076506^{5}^	0.085364^{10}^	0.079128^{6}^
	MSE	$\hat{\alpha }$	0.032397^{1}^	0.036589^{3}^	0.05011^{8}^	0.036558^{2}^	0.046254^{7}^	0.079524^{11}^	0.04182^{5}^	0.038053^{4}^	0.055948^{10}^	0.054929^{9}^	0.044196^{6}^
		$\hat{a}$	0.007507^{1}^	0.0085^{4}^	0.010965^{8}^	0.007986^{2}^	0.010166^{7}^	0.033047^{11}^	0.01181^{10}^	0.008418^{3}^	0.009613^{5}^	0.011403^{9}^	0.009777^{6}^
	MRE	$\hat{\alpha }$	0.056875^{1}^	0.060243^{3}^	0.07127^{8}^	0.059044^{2}^	0.067997^{7}^	0.089903^{11}^	0.063919^{5}^	0.061965^{4}^	0.074768^{10}^	0.074287^{9}^	0.0662^{6}^
		$\hat{a}$	0.046441^{1}^	0.048609^{3}^	0.056339^{8}^	0.047793^{2}^	0.053869^{7}^	0.097133^{11}^	0.056779^{9}^	0.048655^{4}^	0.051004^{5}^	0.056909^{10}^	0.052752^{6}^
	∑*Ranks*		6^{1}^	19^{3}^	48^{9}^	12^{2}^	42^{6}^	66^{11}^	43^{7}^	23^{4}^	45^{8}^	56^{10}^	36^{5}^

**Table 4:** Simulation values of BIAS, MSE and MRE for (*α* = 0.5, *a* = 2.5).

n	Est.	Est. Par.	MLE	ADE	CVME	MPSE	LSE	PCE	RTADE	WLSE	LTADE	MSADE	MSALDE
	BIAS	$\hat{\alpha }$	0.177463^{2}^	0.191461^{5}^	0.227876^{11}^	0.169384^{1}^	0.20694^{8}^	0.219228^{9}^	0.193726^{7}^	0.193527^{6}^	0.226219^{10}^	0.184133^{4}^	0.184092^{3}^
		$\hat{a}$	0.596297^{4}^	0.62115^{6}^	0.813701^{10}^	0.507071^{1}^	0.6527^{7}^	0.829841^{11}^	0.754674^{9}^	0.605885^{5}^	0.664343^{8}^	0.516149^{2}^	0.521017^{3}^
	MSE	$\hat{\alpha }$	0.058177^{3}^	0.067091^{6}^	0.109535^{11}^	0.043501^{1}^	0.072923^{8}^	0.076863^{9}^	0.070496^{7}^	0.064212^{5}^	0.093194^{10}^	0.061375^{4}^	0.055984^{2}^
		$\hat{a}$	0.733561^{4}^	1.195056^{9}^	1.947208^{10}^	0.404055^{1}^	0.932799^{6}^	1.181158^{8}^	2.147357^{11}^	0.793037^{5}^	1.05412^{7}^	0.59964^{3}^	0.501207^{2}^
	MRE	$\hat{\alpha }$	0.354926^{2}^	0.382922^{5}^	0.455752^{11}^	0.338768^{1}^	0.413881^{8}^	0.438456^{9}^	0.387452^{7}^	0.387054^{6}^	0.452438^{10}^	0.368266^{4}^	0.368184^{3}^
		$\hat{a}$	0.238519^{4}^	0.24846^{6}^	0.32548^{10}^	0.202828^{1}^	0.26108^{7}^	0.331937^{11}^	0.30187^{9}^	0.242354^{5}^	0.265737^{8}^	0.20646^{2}^	0.208407^{3}^
	∑*Ranks*		19^{3.5}^	37^{6}^	63^{11}^	6^{1}^	44^{7}^	57^{10}^	50^{8}^	32^{5}^	53^{9}^	19^{3.5}^	16^{2}^
	BIAS	$\hat{\alpha }$	0.108482^{1}^	0.110936^{2}^	0.133566^{9}^	0.113652^{3}^	0.135193^{10}^	0.147553^{11}^	0.123249^{6}^	0.1167^{4}^	0.130941^{7}^	0.131826^{8}^	0.122503^{5}^
		$\hat{a}$	0.348557^{3}^	0.338748^{2}^	0.408709^{9}^	0.336745^{1}^	0.39742^{8}^	0.58739^{11}^	0.414234^{10}^	0.360352^{5}^	0.363309^{7}^	0.362392^{6}^	0.351167^{4}^
	MSE	$\hat{\alpha }$	0.02052^{2}^	0.020589^{3}^	0.03168^{10}^	0.019816^{1}^	0.031329^{9}^	0.033272^{11}^	0.025427^{6}^	0.022727^{5}^	0.029351^{8}^	0.027806^{7}^	0.022586^{4}^
		$\hat{a}$	0.215585^{4}^	0.193232^{2}^	0.318143^{9}^	0.173872^{1}^	0.289262^{8}^	0.539613^{11}^	0.333611^{10}^	0.2273^{5}^	0.234652^{6}^	0.240919^{7}^	0.203153^{3}^
	MRE	$\hat{\alpha }$	0.216964^{1}^	0.221872^{2}^	0.267132^{9}^	0.227303^{3}^	0.270386^{10}^	0.295106^{11}^	0.246497^{6}^	0.233399^{4}^	0.261882^{7}^	0.263652^{8}^	0.245007^{5}^
		$\hat{a}$	0.139423^{3}^	0.135499^{2}^	0.163483^{9}^	0.134698^{1}^	0.158968^{8}^	0.234956^{11}^	0.165694^{10}^	0.144141^{5}^	0.145324^{7}^	0.144957^{6}^	0.140467^{4}^
	∑*Ranks*		14^{3}^	13^{2}^	55^{10}^	10^{1}^	53^{9}^	66^{11}^	48^{8}^	28^{5}^	42^{6.5}^	42^{6.5}^	25^{4}^
	BIAS	$\hat{\alpha }$	0.082146^{1}^	0.092141^{4}^	0.100712^{9}^	0.088212^{2}^	0.099155^{7}^	0.117424^{11}^	0.093542^{5}^	0.088681^{3}^	0.105182^{10}^	0.10045^{8}^	0.096759^{6}^
		$\hat{a}$	0.250142^{1}^	0.280388^{5}^	0.313589^{9}^	0.250908^{2}^	0.293497^{8}^	0.511875^{11}^	0.322982^{10}^	0.261892^{3}^	0.290422^{7}^	0.281917^{6}^	0.275861^{4}^
	MSE	$\hat{\alpha }$	0.010897^{1}^	0.01375^{4}^	0.016304^{9}^	0.01157^{2}^	0.015356^{7}^	0.021409^{11}^	0.014292^{6}^	0.012487^{3}^	0.018134^{10}^	0.015789^{8}^	0.014274^{5}^
		$\hat{a}$	0.109069^{2}^	0.125861^{5}^	0.165015^{9}^	0.097103^{1}^	0.143404^{7}^	0.407632^{11}^	0.18333^{10}^	0.116911^{3}^	0.143592^{8}^	0.134845^{6}^	0.116977^{4}^
	MRE	$\hat{\alpha }$	0.164291^{1}^	0.184283^{4}^	0.201425^{9}^	0.176425^{2}^	0.19831^{7}^	0.234848^{11}^	0.187083^{5}^	0.177362^{3}^	0.210364^{10}^	0.2009^{8}^	0.193517^{6}^
		$\hat{a}$	0.100057^{1}^	0.112155^{5}^	0.125436^{9}^	0.100363^{2}^	0.117399^{8}^	0.20475^{11}^	0.129193^{10}^	0.104757^{3}^	0.116169^{7}^	0.112767^{6}^	0.110344^{4}^
	∑*Ranks*		7^{1}^	27^{4}^	54^{10}^	11^{2}^	44^{7}^	66^{11}^	46^{8}^	18^{3}^	52^{9}^	42^{6}^	29^{5}^
	BIAS	$\hat{\alpha }$	0.056103^{1}^	0.062423^{3}^	0.071511^{8}^	0.058744^{2}^	0.069405^{6}^	0.084695^{11}^	0.065025^{5}^	0.062471^{4}^	0.075915^{10}^	0.075822^{9}^	0.069984^{7}^
		$\hat{a}$	0.170313^{1}^	0.186669^{4}^	0.214952^{9}^	0.177835^{2}^	0.207327^{7}^	0.357109^{11}^	0.225251^{10}^	0.186606^{3}^	0.201307^{5}^	0.214166^{8}^	0.205649^{6}^
	MSE	$\hat{\alpha }$	0.005128^{1}^	0.006419^{4}^	0.008328^{8}^	0.005226^{2}^	0.007511^{6}^	0.011146^{11}^	0.006767^{5}^	0.006206^{3}^	0.00929^{10}^	0.008735^{9}^	0.007654^{7}^
		$\hat{a}$	0.047102^{1}^	0.055908^{3}^	0.078792^{9}^	0.047918^{2}^	0.06935^{7}^	0.198199^{11}^	0.085524^{10}^	0.056028^{4}^	0.067923^{6}^	0.074926^{8}^	0.06498^{5}^
	MRE	$\hat{\alpha }$	0.112205^{1}^	0.124845^{3}^	0.143022^{8}^	0.117488^{2}^	0.138809^{6}^	0.16939^{11}^	0.130051^{5}^	0.124941^{4}^	0.15183^{10}^	0.151643^{9}^	0.139967^{7}^
		$\hat{a}$	0.068125^{1}^	0.074668^{4}^	0.085981^{9}^	0.071134^{2}^	0.082931^{7}^	0.142844^{11}^	0.0901^{10}^	0.074642^{3}^	0.080523^{5}^	0.085666^{8}^	0.082259^{6}^
	∑*Ranks*		6^{1}^	21^{3.5}^	51^{9.5}^	12^{2}^	39^{6}^	66^{11}^	45^{7}^	21^{3.5}^	46^{8}^	51^{9.5}^	38^{5}^
	BIAS	$\hat{\alpha }$	0.046808^{1}^	0.050644^{3}^	0.056187^{7}^	0.049051^{2}^	0.057479^{8}^	0.073869^{11}^	0.051232^{4}^	0.051418^{5}^	0.062398^{10}^	0.05888^{9}^	0.052576^{6}^
		$\hat{a}$	0.141459^{1}^	0.147861^{3}^	0.168811^{7}^	0.143989^{2}^	0.169403^{8}^	0.319571^{11}^	0.17322^{10}^	0.156242^{4}^	0.164001^{6}^	0.169983^{9}^	0.161707^{5}^
	MSE	$\hat{\alpha }$	0.003444^{1}^	0.004049^{3}^	0.005047^{7}^	0.003765^{2}^	0.005388^{8}^	0.008378^{11}^	0.004266^{5}^	0.004129^{4}^	0.006195^{10}^	0.005441^{9}^	0.004303^{6}^
		$\hat{a}$	0.031472^{1}^	0.034578^{3}^	0.046124^{7}^	0.033041^{2}^	0.046392^{8}^	0.156322^{11}^	0.048677^{10}^	0.038019^{4}^	0.042677^{6}^	0.047009^{9}^	0.041157^{5}^
	MRE	$\hat{\alpha }$	0.093616^{1}^	0.101287^{3}^	0.112374^{7}^	0.098101^{2}^	0.114957^{8}^	0.147739^{11}^	0.102465^{4}^	0.102837^{5}^	0.124797^{10}^	0.117761^{9}^	0.105152^{6}^
		$\hat{a}$	0.056583^{1}^	0.059144^{3}^	0.067524^{7}^	0.057596^{2}^	0.067761^{8}^	0.127828^{11}^	0.069288^{10}^	0.062497^{4}^	0.0656^{6}^	0.067993^{9}^	0.064683^{5}^
	∑*Ranks*		6^{1}^	18^{3}^	42^{6}^	12^{2}^	48^{8.5}^	66^{11}^	43^{7}^	26^{4}^	48^{8.5}^	54^{10}^	33^{5}^
	BIAS	$\hat{\alpha }$	0.035233^{1}^	0.041144^{5}^	0.046497^{9}^	0.036526^{2}^	0.04339^{7}^	0.055356^{11}^	0.04034^{4}^	0.040268^{3}^	0.048157^{10}^	0.046368^{8}^	0.04252^{6}^
		$\hat{a}$	0.112704^{2}^	0.121588^{4}^	0.138791^{10}^	0.104448^{1}^	0.12917^{7}^	0.250242^{11}^	0.133608^{8}^	0.116489^{3}^	0.125087^{5}^	0.137228^{9}^	0.126725^{6}^
	MSE	$\hat{\alpha }$	0.001968^{1}^	0.002701^{5}^	0.003506^{9}^	0.002016^{2}^	0.002982^{7}^	0.004806^{11}^	0.002555^{4}^	0.002467^{3}^	0.003613^{10}^	0.003411^{8}^	0.002787^{6}^
		$\hat{a}$	0.019744^{2}^	0.024632^{5}^	0.029884^{9}^	0.017786^{1}^	0.026522^{7}^	0.097721^{11}^	0.028088^{8}^	0.021178^{3}^	0.025287^{6}^	0.030779^{10}^	0.024547^{4}^
	MRE	$\hat{\alpha }$	0.070465^{1}^	0.082288^{5}^	0.092994^{9}^	0.073051^{2}^	0.086779^{7}^	0.110711^{11}^	0.080681^{4}^	0.080537^{3}^	0.096315^{10}^	0.092736^{8}^	0.08504^{6}^
		$\hat{a}$	0.045082^{2}^	0.048635^{4}^	0.055516^{10}^	0.041779^{1}^	0.051668^{7}^	0.100097^{11}^	0.053443^{8}^	0.046596^{3}^	0.050035^{5}^	0.054891^{9}^	0.05069^{6}^
	∑*Ranks*		9^{1.5}^	28^{4}^	56^{10}^	9^{1.5}^	42^{7}^	66^{11}^	36^{6}^	18^{3}^	46^{8}^	52^{9}^	34^{5}^

**Table 5:** Simulation values of BIAS, MSE and MRE for (*α* = 3, *a* = 4).

n	Est.	Est. Par.	MLE	ADE	CVME	MPSE	LSE	PCE	RTADE	WLSE	LTADE	MSADE	MSALDE
	BIAS	$\hat{\alpha }$	0.679389^{3}^	0.672482^{1}^	0.806945^{11}^	0.674966^{2}^	0.755749^{7}^	0.783868^{10}^	0.762392^{8}^	0.70514^{4}^	0.778775^{9}^	0.71373^{5}^	0.722868^{6}^
		$\hat{a}$	1.33309^{6}^	1.227139^{4}^	1.776382^{10}^	1.089366^{1}^	1.439147^{9}^	1.404214^{7}^	1.890905^{11}^	1.32838^{5}^	1.438843^{8}^	1.181281^{3}^	1.138545^{2}^
	MSE	$\hat{\alpha }$	0.819934^{4}^	0.777124^{2}^	1.189851^{11}^	0.664387^{1}^	0.965457^{7}^	0.978338^{8}^	1.033229^{9}^	0.86081^{6}^	1.043361^{10}^	0.820633^{5}^	0.792179^{3}^
		$\hat{a}$	4.239991^{6}^	3.400627^{4}^	11.037634^{10}^	1.904351^{1}^	5.93256^{9}^	3.560397^{5}^	18.626633^{11}^	4.612968^{7}^	4.915083^{8}^	3.134863^{3}^	2.25588^{2}^
	MRE	$\hat{\alpha }$	0.226463^{3}^	0.224161^{1}^	0.268982^{11}^	0.224989^{2}^	0.251916^{7}^	0.261289^{10}^	0.254131^{8}^	0.235047^{4}^	0.259592^{9}^	0.23791^{5}^	0.240956^{6}^
		$\hat{a}$	0.333272^{6}^	0.306785^{4}^	0.444095^{10}^	0.272342^{1}^	0.359787^{9}^	0.351053^{7}^	0.472726^{11}^	0.332095^{5}^	0.359711^{8}^	0.29532^{3}^	0.284636^{2}^
	∑*Ranks*		30^{5}^	18^{2}^	55^{11}^	8^{1}^	50^{8.5}^	49^{7}^	50^{8.5}^	33^{6}^	54^{10}^	26^{4}^	23^{3}^
	BIAS	$\hat{\alpha }$	0.410925^{1}^	0.438118^{4}^	0.494907^{10}^	0.429939^{3}^	0.488172^{9}^	0.535343^{11}^	0.464754^{6}^	0.428901^{2}^	0.483066^{8}^	0.471674^{7}^	0.458472^{5}^
		$\hat{a}$	0.705744^{2}^	0.786619^{6}^	0.955617^{10}^	0.688694^{1}^	0.842142^{8}^	0.96431^{11}^	0.921741^{9}^	0.756028^{4}^	0.781127^{5}^	0.788992^{7}^	0.728161^{3}^
	MSE	$\hat{\alpha }$	0.2788^{2}^	0.308398^{4}^	0.405883^{10}^	0.275332^{1}^	0.383062^{9}^	0.433457^{11}^	0.345337^{6}^	0.296296^{3}^	0.382208^{8}^	0.365178^{7}^	0.316051^{5}^
		$\hat{a}$	0.923369^{3}^	1.215158^{7}^	1.930207^{11}^	0.730839^{1}^	1.262549^{8}^	1.576396^{9}^	1.58999^{10}^	1.071024^{4}^	1.133921^{5}^	1.202663^{6}^	0.819734^{2}^
	MRE	$\hat{\alpha }$	0.136975^{1}^	0.146039^{4}^	0.164969^{10}^	0.143313^{3}^	0.162724^{9}^	0.178448^{11}^	0.154918^{6}^	0.142967^{2}^	0.161022^{8}^	0.157225^{7}^	0.152824^{5}^
		$\hat{a}$	0.176436^{2}^	0.196655^{6}^	0.238904^{10}^	0.172173^{1}^	0.210535^{8}^	0.241078^{11}^	0.230435^{9}^	0.189007^{4}^	0.195282^{5}^	0.197248^{7}^	0.18204^{3}^
	∑*Ranks*		11^{2}^	31^{5}^	61^{10}^	10^{1}^	51^{9}^	64^{11}^	46^{8}^	19^{3}^	39^{6}^	41^{7}^	23^{4}^
	BIAS	$\hat{\alpha }$	0.298747^{1}^	0.339514^{3}^	0.367271^{7}^	0.322065^{2}^	0.359612^{6}^	0.423964^{11}^	0.356811^{5}^	0.343573^{4}^	0.37907^{9}^	0.399231^{10}^	0.367452^{8}^
		$\hat{a}$	0.523188^{2}^	0.550538^{3}^	0.657166^{9}^	0.512555^{1}^	0.624581^{7}^	0.773039^{11}^	0.682018^{10}^	0.588021^{5}^	0.602452^{6}^	0.635227^{8}^	0.586786^{4}^
	MSE	$\hat{\alpha }$	0.142116^{1}^	0.181013^{3}^	0.230408^{8}^	0.154677^{2}^	0.198941^{5}^	0.279076^{11}^	0.211703^{7}^	0.192388^{4}^	0.233488^{9}^	0.258543^{10}^	0.204722^{6}^
		$\hat{a}$	0.44904^{2}^	0.519922^{3}^	0.82487^{10}^	0.397969^{1}^	0.645068^{7}^	0.944882^{11}^	0.82132^{9}^	0.60228^{5}^	0.643889^{6}^	0.730263^{8}^	0.525867^{4}^
	MRE	$\hat{\alpha }$	0.099582^{1}^	0.113171^{3}^	0.122424^{7}^	0.107355^{2}^	0.119871^{6}^	0.141321^{11}^	0.118937^{5}^	0.114524^{4}^	0.126357^{9}^	0.133077^{10}^	0.122484^{8}^
		$\hat{a}$	0.130797^{2}^	0.137634^{3}^	0.164292^{9}^	0.128139^{1}^	0.156145^{7}^	0.19326^{11}^	0.170504^{10}^	0.147005^{5}^	0.150613^{6}^	0.158807^{8}^	0.146696^{4}^
	∑*Ranks*		9^{1.5}^	18^{3}^	50^{9}^	9^{1.5}^	38^{6}^	66^{11}^	46^{8}^	27^{4}^	45^{7}^	54^{10}^	34^{5}^
	BIAS	$\hat{\alpha }$	0.224158^{1}^	0.227471^{2}^	0.27132^{8}^	0.227801^{3}^	0.258813^{6}^	0.305866^{11}^	0.250523^{5}^	0.233597^{4}^	0.272379^{10}^	0.271944^{9}^	0.260204^{7}^
		$\hat{a}$	0.366514^{2}^	0.385532^{3}^	0.457061^{9}^	0.358637^{1}^	0.449386^{8}^	0.585061^{11}^	0.479351^{10}^	0.398427^{4}^	0.436038^{7}^	0.434917^{6}^	0.424392^{5}^
	MSE	$\hat{\alpha }$	0.082182^{3}^	0.081137^{2}^	0.113436^{8}^	0.079351^{1}^	0.106791^{7}^	0.144514^{11}^	0.099434^{5}^	0.084374^{4}^	0.119252^{10}^	0.115336^{9}^	0.105119^{6}^
		$\hat{a}$	0.226617^{2}^	0.239277^{3}^	0.35361^{9}^	0.204299^{1}^	0.331595^{8}^	0.521269^{11}^	0.383037^{10}^	0.264577^{4}^	0.316757^{7}^	0.314461^{6}^	0.282668^{5}^
	MRE	$\hat{\alpha }$	0.074719^{1}^	0.075824^{2}^	0.09044^{8}^	0.075934^{3}^	0.086271^{6}^	0.101955^{11}^	0.083508^{5}^	0.077866^{4}^	0.090793^{10}^	0.090648^{9}^	0.086735^{7}^
		$\hat{a}$	0.091628^{2}^	0.096383^{3}^	0.114265^{9}^	0.089659^{1}^	0.112347^{8}^	0.146265^{11}^	0.119838^{10}^	0.099607^{4}^	0.10901^{7}^	0.108729^{6}^	0.106098^{5}^
	∑*Ranks*		11^{2}^	15^{3}^	51^{9.5}^	10^{1}^	43^{6}^	66^{11}^	45^{7.5}^	24^{4}^	51^{9.5}^	45^{7.5}^	35^{5}^
	BIAS	$\hat{\alpha }$	0.178099^{2}^	0.191107^{3}^	0.202875^{5}^	0.175808^{1}^	0.20473^{6}^	0.262468^{11}^	0.211408^{8}^	0.195105^{4}^	0.218768^{9}^	0.225325^{10}^	0.209099^{7}^
		$\hat{a}$	0.290726^{2}^	0.338214^{4}^	0.349143^{6}^	0.277732^{1}^	0.361564^{8}^	0.510798^{11}^	0.394269^{10}^	0.328312^{3}^	0.351022^{7}^	0.377094^{9}^	0.346419^{5}^
	MSE	$\hat{\alpha }$	0.050226^{2}^	0.05958^{4}^	0.066825^{5}^	0.048251^{1}^	0.06733^{6}^	0.105982^{11}^	0.071879^{8}^	0.059121^{3}^	0.077105^{9}^	0.079636^{10}^	0.067553^{7}^
		$\hat{a}$	0.137231^{2}^	0.182677^{4}^	0.199189^{6}^	0.128479^{1}^	0.212591^{8}^	0.396841^{11}^	0.256548^{10}^	0.170429^{3}^	0.204721^{7}^	0.227815^{9}^	0.188698^{5}^
	MRE	$\hat{\alpha }$	0.059366^{2}^	0.063702^{3}^	0.067625^{5}^	0.058603^{1}^	0.068243^{6}^	0.087489^{11}^	0.070469^{8}^	0.065035^{4}^	0.072923^{9}^	0.075108^{10}^	0.0697^{7}^
		$\hat{a}$	0.072681^{2}^	0.084554^{4}^	0.087286^{6}^	0.069433^{1}^	0.090391^{8}^	0.127699^{11}^	0.098567^{10}^	0.082078^{3}^	0.087755^{7}^	0.094273^{9}^	0.086605^{5}^
	∑*Ranks*		12^{2}^	22^{4}^	33^{5}^	6^{1}^	42^{7}^	66^{11}^	54^{9}^	20^{3}^	48^{8}^	57^{10}^	36^{6}^
	BIAS	$\hat{\alpha }$	0.136129^{2}^	0.149044^{4}^	0.170932^{9}^	0.130625^{1}^	0.159677^{6}^	0.199614^{11}^	0.157937^{5}^	0.146281^{3}^	0.169687^{8}^	0.176743^{10}^	0.162948^{7}^
		$\hat{a}$	0.23199^{2}^	0.243083^{3}^	0.293184^{10}^	0.203672^{1}^	0.276736^{7}^	0.392015^{11}^	0.29299^{9}^	0.249042^{4}^	0.268105^{6}^	0.2796^{8}^	0.25873^{5}^
	MSE	$\hat{\alpha }$	0.029514^{2}^	0.0347^{4}^	0.046061^{9}^	0.028031^{1}^	0.040438^{6}^	0.062798^{11}^	0.039357^{5}^	0.034517^{3}^	0.044847^{8}^	0.049262^{10}^	0.041373^{7}^
		$\hat{a}$	0.086069^{2}^	0.094464^{3}^	0.136535^{9}^	0.074091^{1}^	0.122356^{7}^	0.238836^{11}^	0.138654^{10}^	0.10492^{4}^	0.115985^{6}^	0.127582^{8}^	0.106968^{5}^
	MRE	$\hat{\alpha }$	0.045376^{2}^	0.049681^{4}^	0.056977^{9}^	0.043542^{1}^	0.053226^{6}^	0.066538^{11}^	0.052646^{5}^	0.04876^{3}^	0.056562^{8}^	0.058914^{10}^	0.054316^{7}^
		$\hat{a}$	0.057998^{2}^	0.060771^{3}^	0.073296^{10}^	0.050918^{1}^	0.069184^{7}^	0.098004^{11}^	0.073247^{9}^	0.06226^{4}^	0.067026^{6}^	0.0699^{8}^	0.064682^{5}^
	∑*Ranks*		12^{2}^	21^{3.5}^	56^{10}^	6^{1}^	39^{6}^	66^{11}^	43^{8}^	21^{3.5}^	42^{7}^	54^{9}^	36^{5}^

**Table 6:** Partial and overall ranks of all the methods of estimation of WBHD by various values of model parameters.

Parameter	*n*	MLE	ADE	CVME	MPSE	LSE	PCE	RTADE	WLSE	LTADE	MSADE	MSALDE
*α* = 0.75, *a* = 0.25	25	3.0	5.0	11.0	1.0	8.0	6.0	7.0	9.0	10.0	4.0	2.0
	60	4.0	3.0	11.0	1.0	8.0	9.0	7.0	6.0	10.0	5.0	2.0
	100	2.0	3.0	8.0	1.0	10.0	11.0	7.0	5.5	9.0	5.5	4.0
	200	2.0	3.0	8.0	1.0	9.0	10.0	6.0	5.0	11.0	7.0	4.0
	300	2.0	4.0	10.5	1.0	7.5	10.5	6.0	4.0	9.0	7.5	4.0
	500	2.0	3.0	9.0	1.0	10.0	11.0	6.5	5.0	8.0	6.5	4.0
*α* = 1.5, *a* = 0.75	25	3.0	4.0	11.0	1.0	7.0	10.0	8.5	6.0	8.5	5.0	2.0
	60	2.0	5.0	9.0	1.0	8.0	11.0	6.0	3.0	10.0	7.0	4.0
	100	1.5	3.0	10.0	1.5	7.0	11.0	6.0	5.0	8.0	9.0	4.0
	200	2.0	1.0	8.5	3.0	7.0	11.0	6.0	4.0	10.0	8.5	5.0
	300	1.0	4.0	8.5	2.0	8.5	11.0	6.0	3.0	7.0	10.0	5.0
	500	2.0	4.0	9.0	1.0	7.0	11.0	6.0	3.0	8.0	10.0	5.0
*α* = 2.5, *a* = 1.5	25	5.0	3.0	11.0	1.0	7.0	10.0	9.0	6.0	8.0	2.0	4.0
	60	3.0	2.0	10.0	1.0	6.0	11.0	7.0	4.0	9.0	8.0	5.0
	100	2.0	3.0	9.0	1.0	7.0	11.0	8.0	4.0	5.0	10.0	6.0
	200	1.0	3.0	6.5	2.0	8.0	11.0	6.5	4.0	9.0	10.0	5.0
	300	1.5	3.5	7.0	1.5	9.0	11.0	8.0	3.5	6.0	10.0	5.0
	500	1.0	3.0	9.0	2.0	6.0	11.0	7.0	4.0	8.0	10.0	5.0
*α* = 0.5, *a* = 2.5	25	3.5	6.0	11.0	1.0	7.0	10.0	8.0	5.0	9.0	3.5	2.0
	60	3.0	2.0	10.0	1.0	9.0	11.0	8.0	5.0	6.5	6.5	4.0
	100	1.0	4.0	10.0	2.0	7.0	11.0	8.0	3.0	9.0	6.0	5.0
	200	1.0	3.5	9.5	2.0	6.0	11.0	7.0	3.5	8.0	9.5	5.0
	300	1.0	3.0	6.0	2.0	8.5	11.0	7.0	4.0	8.5	10.0	5.0
	500	1.5	4.0	10.0	1.5	7.0	11.0	6.0	3.0	8.0	9.0	5.0
*α* = 3, *a* = 4	25	5.0	2.0	11.0	1.0	8.5	7.0	8.5	6.0	10.0	4.0	3.0
	60	2.0	5.0	10.0	1.0	9.0	11.0	8.0	3.0	6.0	7.0	4.0
	100	1.5	3.0	9.0	1.5	6.0	11.0	8.0	4.0	7.0	10.0	5.0
	200	2.0	3.0	9.5	1.0	6.0	11.0	7.5	4.0	9.5	7.5	5.0
	300	2.0	4.0	5.0	1.0	7.0	11.0	9.0	3.0	8.0	10.0	6.0
	500	2.0	3.5	10.0	1.0	6.0	11.0	8.0	3.5	7.0	9.0	5.0
∑ Ranks		65.5	102.5	277.0	40.0	227.0	314.5	216.5	131.0	250.0	227.0	129.0
Overall Rank		2.0	3.0	10.0	1.0	7.5	11.0	6.0	5.0	9.0	7.5	4.0

### 4.3 Concluding remarks on simulation results

1. After recording the results we found that as the sample size get larger the MSE diminishes gradually
2. After recording the results we found that as the sample size get larger the MRE diminishes gradually
3. After recording the results we found that as the sample size get larger the BIAS diminishes gradually
4. By referring to Table 6 we can see that the best estimation method is the MPSE as it has the lowest overall rank.
5. By referring to Table 6 we can see that the second best estimation method is the MLE as it has the second lowest overall rank.

## 5 Risk measures

In this section we study some risk measures for WBHD. One of this measures is value at risk (VR) which refers to to a quantitative total of the cumulative loss distribution (see Artzner [21]). It is defined for WBHD as follows

$$\begin{array}{c} VR_{q}=\frac{W\left(-\frac{e^{\alpha }\alpha }{{\left(2^{q}-1\right)}^{1/a}-1}\right)-\alpha }{\alpha }. \end{array}$$

The second risk measure is called tail value at risk which is used to estimate the worth of a prospective loss when an event occurs outside of the predetermined probability and it is defined for WBHD as follows

$$\begin{array}{cc} TVR_{q} & =\frac{1}{\left(1-q\right)}\int_{VaR_{q}}^{\infty }x\;\;f\left(x\right)dx=\\ & =. \end{array}$$

### 5.1 Numerical simulations for risk measures

**Fig 3:**
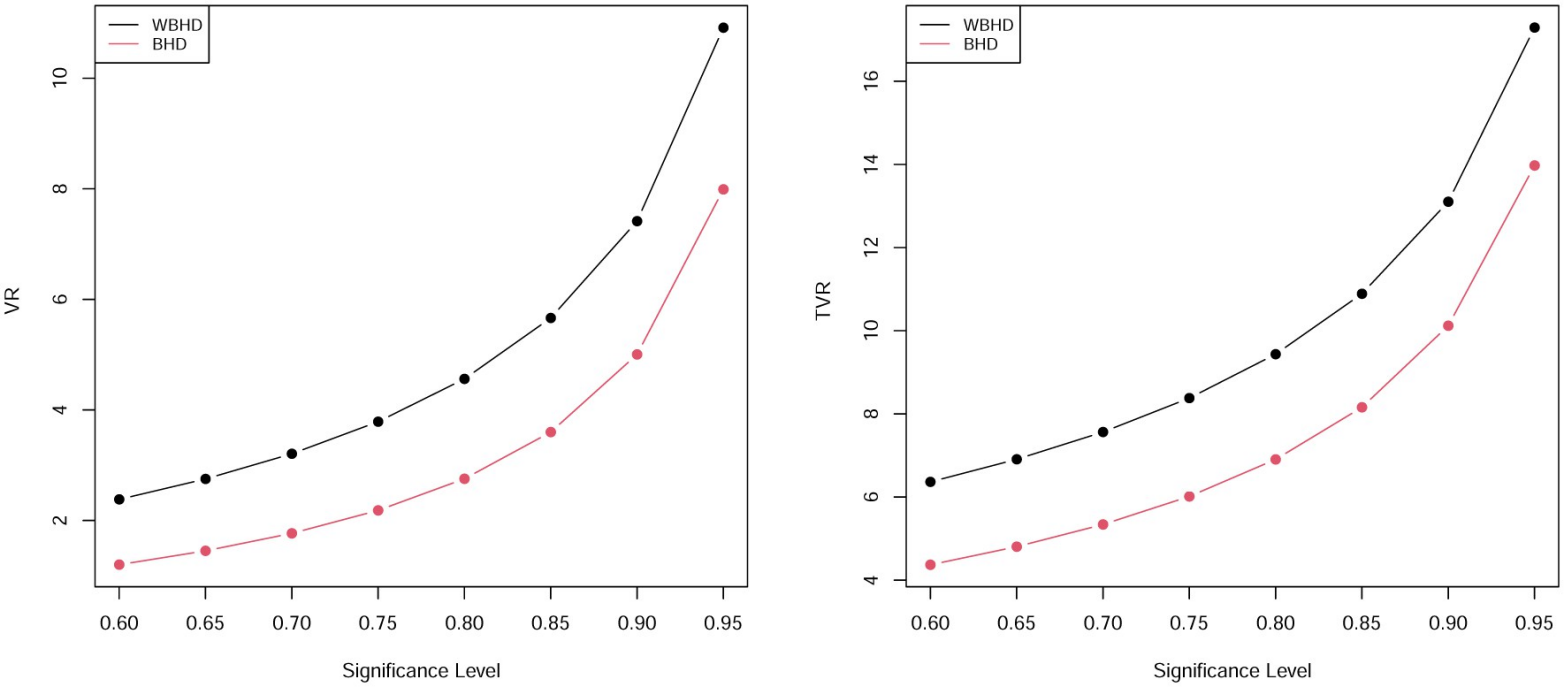
Graphs of the VR and TVR by the values in Table 7.

**Fig 4:**
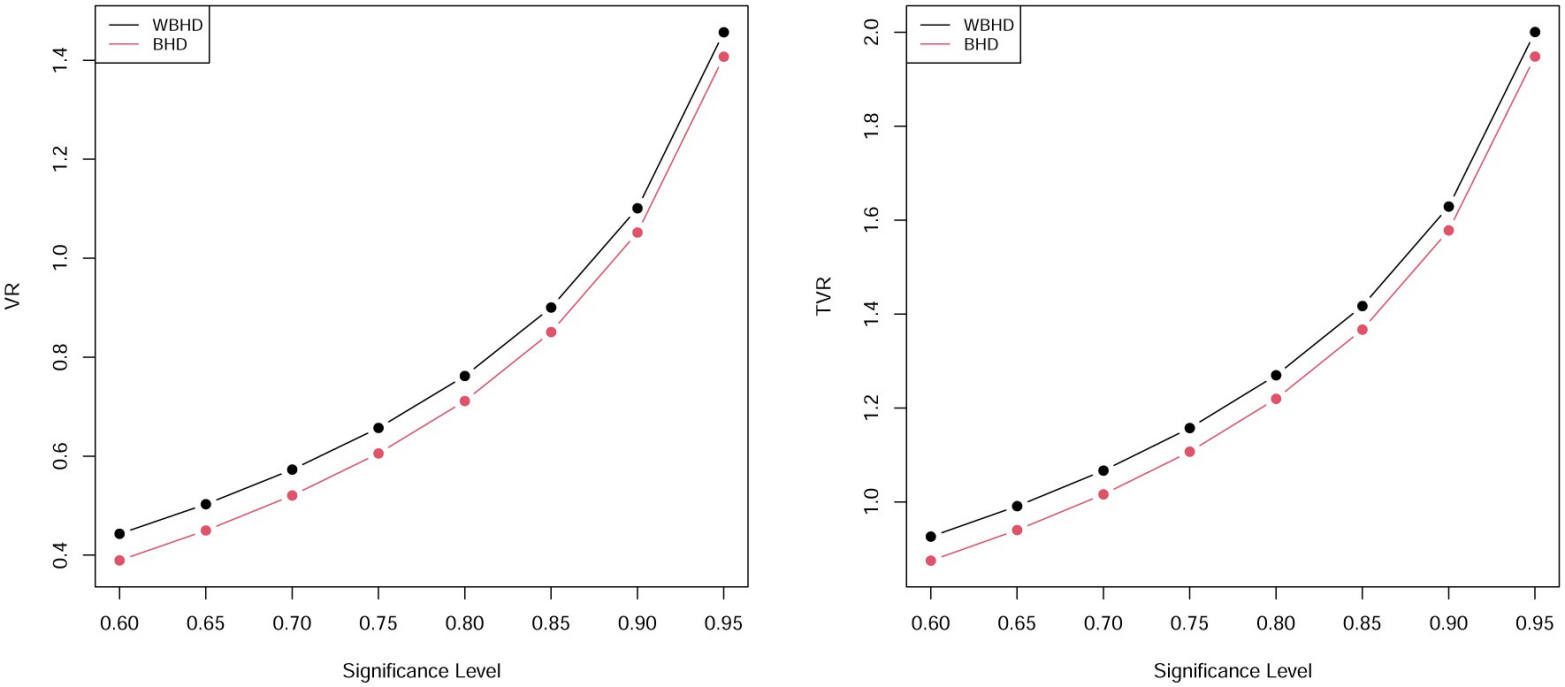
Graphs of the VR and TVR by the values in Table 8.

In this subsection some results for risk measures for WBHD and BHD are discussed. Tables 7 and 8 presented numerical values of the two risk measures which are determined for both of WBHD and BHD, also, these results are presented graphically in in Figs 3 and 4. From these tables, we conclude that our proposed model have larger values for the two measures compared with BHD, so we can say that the WBHD fits heavy tailed model than BHD and it can be used for modeling insurance data set and other heavy tailed real data sets.

**Table 7:** Numerical results of VR and TVR for the WBHD and BHD.

Distribution	parameter	Significance level	VR	TVR
WBHD	*α* = 0.1, *a* = 2.5	0.60	2.38101	6.36337
		0.65	2.75071	6.90673
		0.70	3.20631	7.56284
		0.75	3.78680	8.37862
		0.80	4.56058	9.43499
		0.85	5.66263	10.88808
		0.90	7.41397	13.10199
		0.95	10.91377	17.29114
BHD	*α* = 0.1	0.60	1.20050	4.36933
		0.65	1.45037	4.80483
		0.70	1.76687	5.33864
		0.75	2.18204	6.01349
		0.80	2.75355	6.90406
		0.85	3.59834	8.15693
		0.90	5.00342	10.11918
		0.95	7.98955	13.97313

**Table 8:** Numerical results of VR and TVR for the WBHD and BHD.

Distribution	parameter	Significance level	VR	TVR
WBHD	*α* = 1.5, *a* = 1.5	0.60	0.44295	0.92602
		0.65	0.50278	0.99086
		0.70	0.57277	1.06654
		0.75	0.65691	1.15715
		0.80	0.76187	1.26963
		0.85	0.90028	1.41701
		0.90	1.10083	1.62887
		0.95	1.45648	2.00070
BHD	*α* = 1.5	0.60	0.38926	0.87478
		0.65	0.44966	0.93993
		0.70	0.52041	1.01591
		0.75	0.60542	1.10679
		0.80	0.71131	1.21943
		0.85	0.85062	1.36678
		0.90	1.05180	1.57818
		0.95	1.40709	1.94838

## 6 Analysis of COVID-19 real data sets

The usage of COVID-19 actual world data sets in this section demonstrates the distribution’s adaptability. The first data set provides the COVID-19 mortality rate from Saudi Arabia for a period of forty days, from the 22nd of July to the 30th of August 2021. The second real data set on the mortality rate COVID-19 statistics belongs to Saudi Arabia and covers a period of 32 days, which is recorded from the 15th of September 2020 to the 16th of October 2020. Both of the two real data sets are available at https://covid19.who.int/. We will examine WBHD in contrast to a variety of well-known models, such as Burr-Hatke distribution (BHD) [22], inverse power Burr-Hatke distribution (IPBHD) [23], logarithmic Burr-Hatke exponential distribution (LBHED) [24], alpha power exponential distribution (APED) [25], Frechet distribution (FD), exponential distribution (ED), Lindley distribution (LND), Lomax distribution (LD), Frechet Weibull distribution (FWD) [15], and Maxwell distribution (MD), in order to show how flexible WBHD is.

We make use of a variety of analytical criteria in order to identify which model is the most suited to employ with the COVID-19 actual data sets. These criteria are Akaike information criterion (*A*_1_), the correct Akaike information criterion (*A*_2_), Bayesian information criterion (*A*_3_), Hannan information criterion (*A*_4_). We also consider other information on the model’s overall goodness-of-fit, including Anderson Darling (*F*_1_), Cramer-von Mises (*F*_2_), and Kolmogorov-Smirnov (*F*_3_) with its p-value (*F*_3_(*p*)). The best model for fitting the COVID-19 real data sets is the one with the smallest values of these measures, with the exception of *G*3(*p*) the model with large value is the best model.

**Fig 5:**
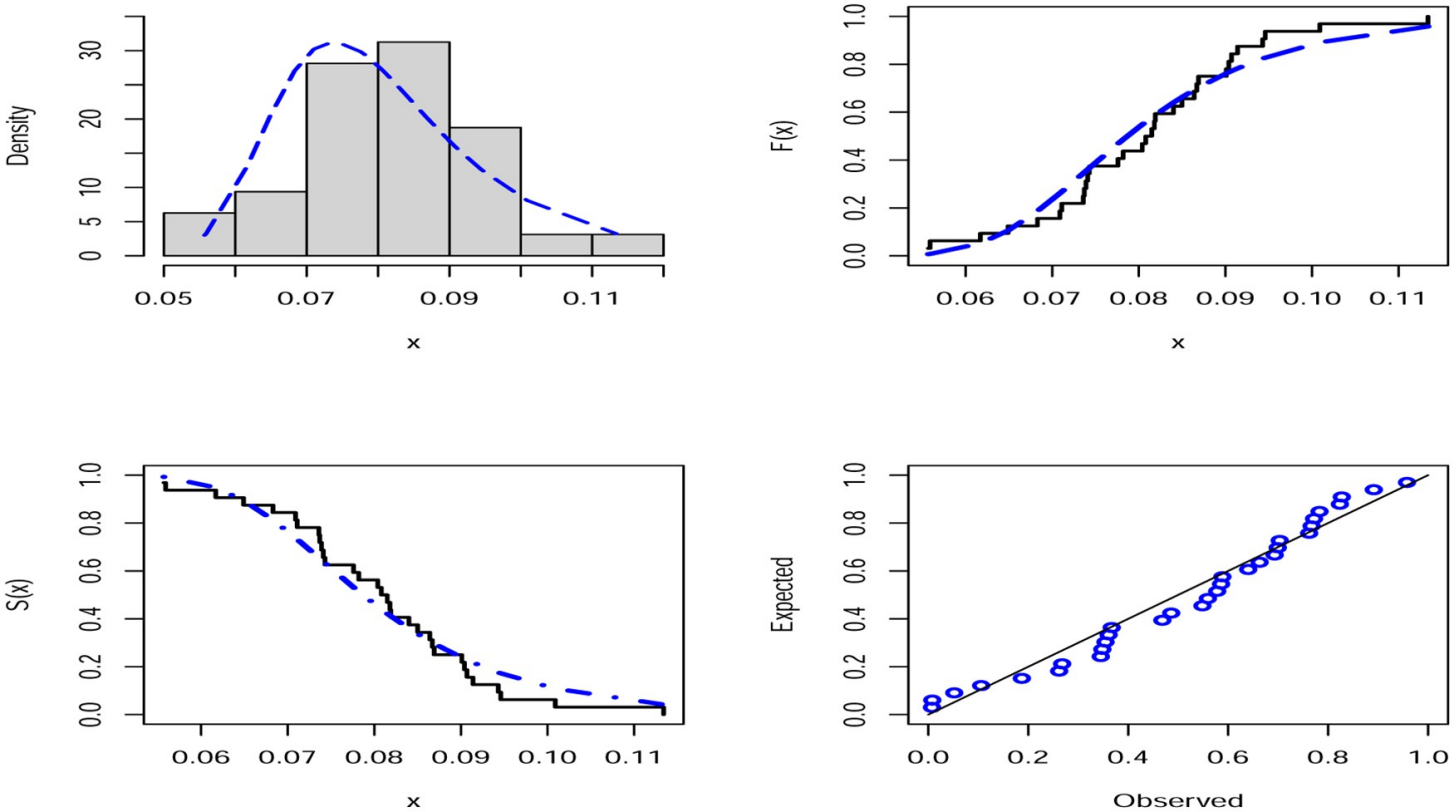
Histogram of the first COVID-19 real data set with the fitted PDF, CDF, SF and P-P plots.

**Fig 6:**
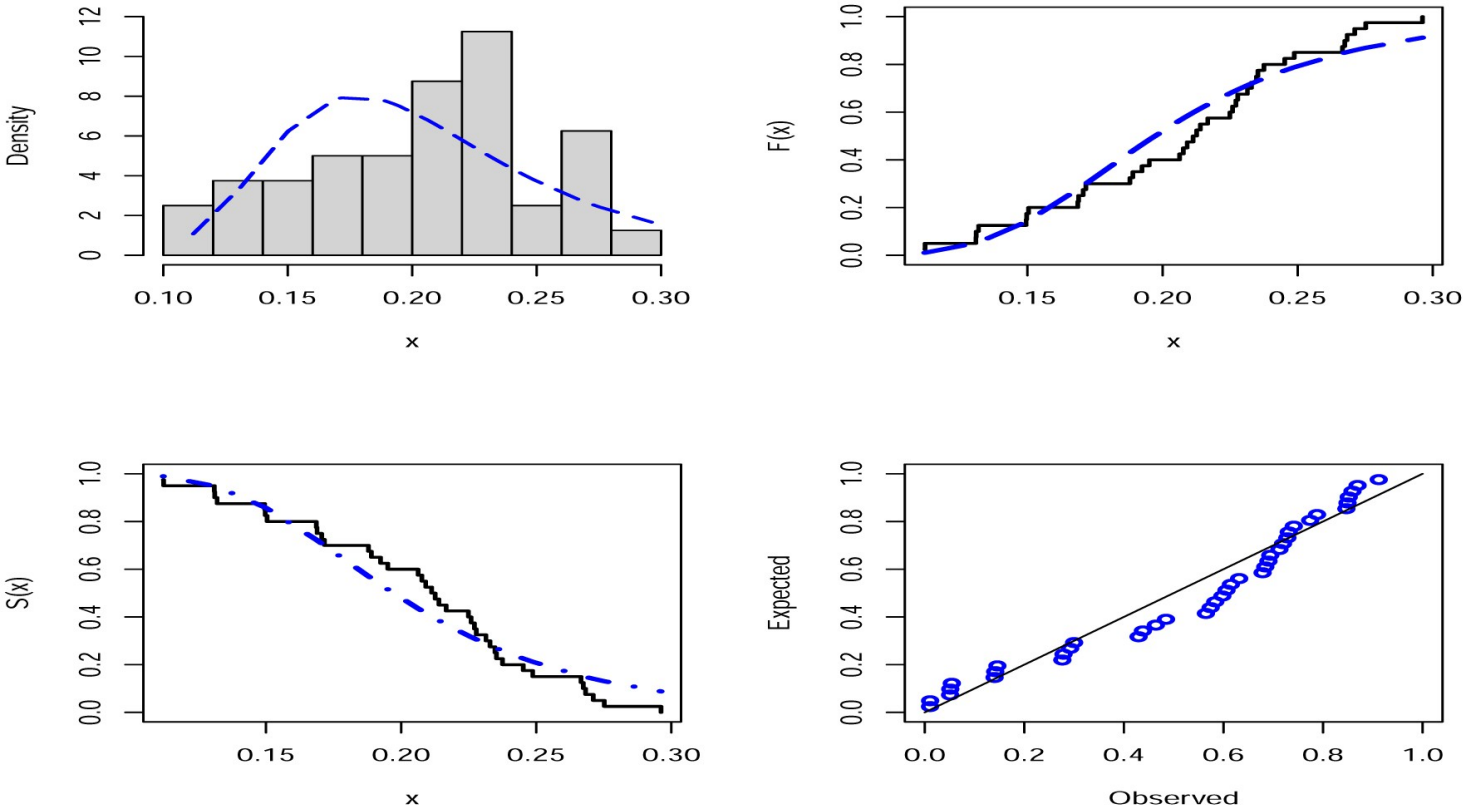
Histogram of the second COVID-19 real data set with the fitted PDF, CDF, SF and P-P plots.

**Fig 7:**
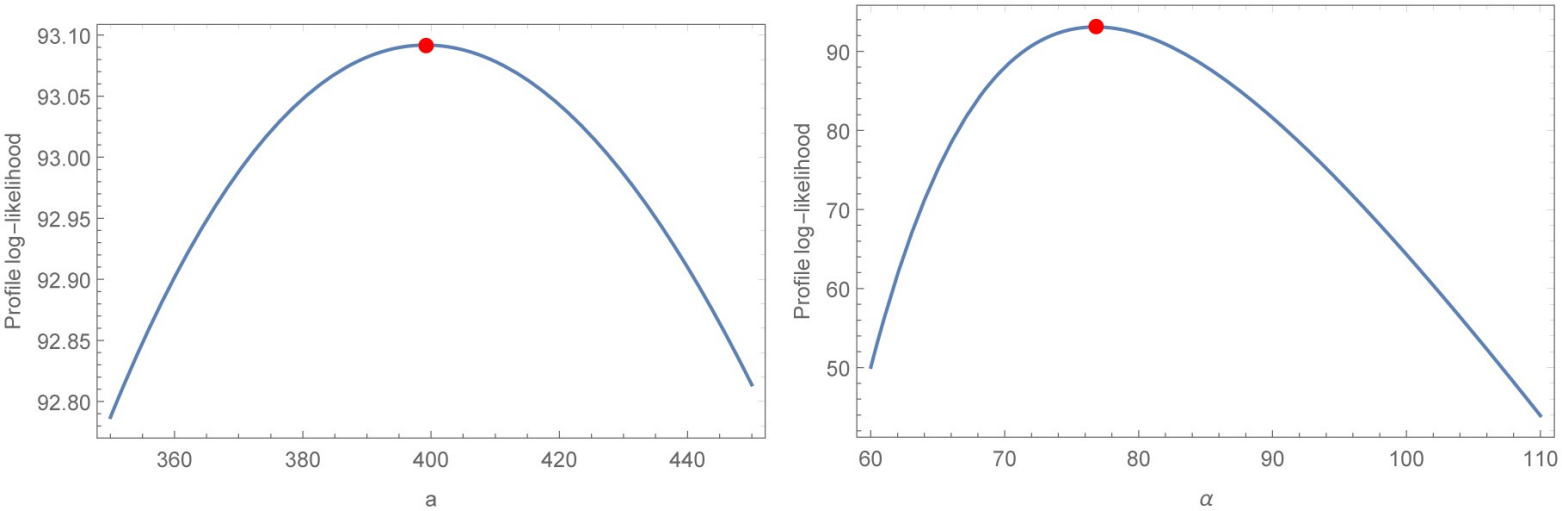
This figure discuses behavior of the log-likelihood function with *a* and *α* parameters of the first COVID-19 real data set. Which proves that the roots are global maximum.

**Fig 8:**
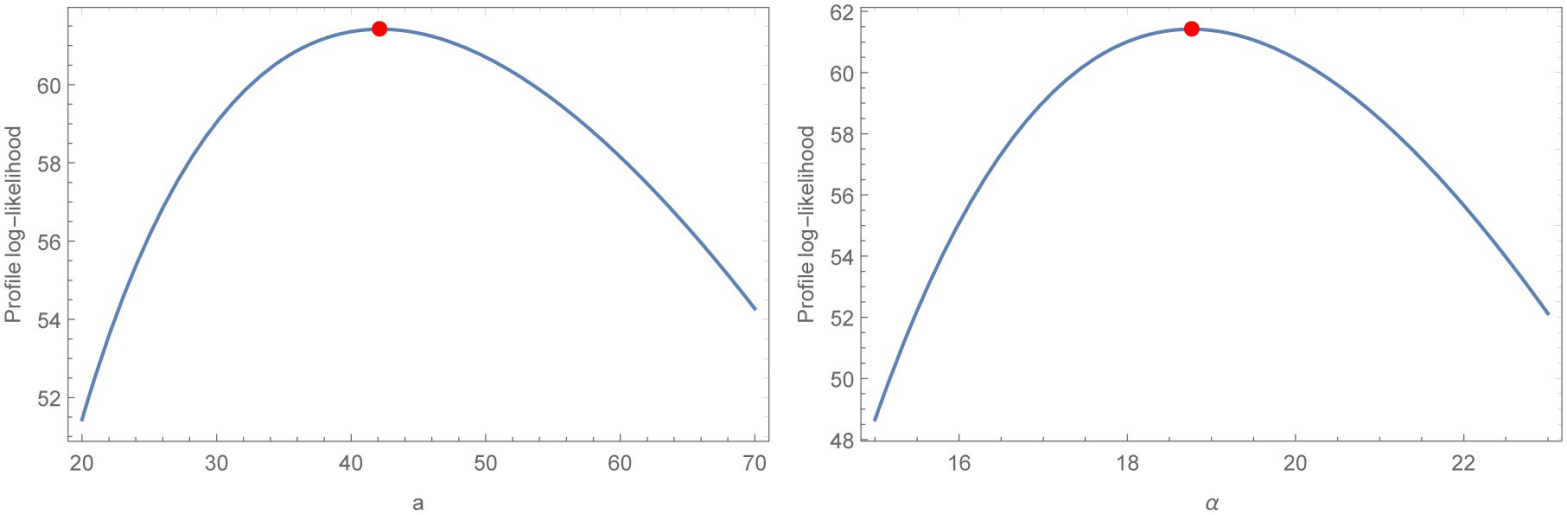
This figure discuses the behavior of the log-likelihood functions with *a* and *α* parameters of the second COVID-19 real data set. Which proves that the roots are global maximum.

For the two COVID-19 actual data sets that were taken into consideration for assessment, analytical measurements as well as MLE estimations and their accompanying standard errors (SE) are provided, respectively, in Tables 9 and 10. As a direct result of this, we could arrive at the conclusion that the WBHD model performs far better than the other models that are comparable to it. The two COVID-19 actual data sets that are shown in Figs 5 and 6, respectively, are fitted with WBHD using the P-P plot as well as the fitted PDF, CDF, and SF plots. Figs 7 and 8 demonstrate, respectively, for the two COVID-19 real data sets, the behavior of the log-likelihood function with estimated parameters, which is a unimodal function for each value of the estimated parameters.

**Table 9:** Numerical values for analyzing the first COVID-19 real data set.

Model	−L	*I*_1_	*I*_2_	*I*_3_	*I*_4_	*F*_1_	*F*_2_	*F*_3_	*F*_3_(*p*)	Est. parameters (SEs)
WBHD	-93.0918	-182.184	-181.77	-179.252	-181.212	0.712869	0.10394	0.126309	0.686965	$\hat{a}=399.159\;\left(290.488\right)$
										$\hat{\alpha }=76.7804\;\left(10.3509\right)$
BHD	-48.5441	-95.0881	-94.9548	-93.6224	-94.6023	10.7389	2.29959	0.500833	<0.00001	$\hat{a}=11.5011\;\left(2.19675\right)$
IPBHD	-3.93522	-3.87043	-3.45664	-0.938962	-2.89873	31.5761	6.27548	0.813049	<0.00001	$\hat{\alpha }=0.040474\;\left(0.0424118\right)$
										$\hat{\eta }=0.590983\;\left(0.0840706\right)$
LBHED	-80.7517	-159.503	-159.37	-158.038	-159.018	38.7578	7.08839	0.809566	<0.00001	$\hat{\alpha }=17.5388\;\left(2.8656\right)$
APED	-84.5976	-165.195	-164.781	-162.264	-164.223	2.90832	0.493393	0.237589	0.0539557	$\hat{\alpha }=5.01409\times 10^{11}\;\left(9.8693\times 10^{11}\right)$
										$\hat{a}=45.7105\;\left(2.66765\right)$
FD	-91.0456	-178.091	-177.677	-175.16	-177.12	1.13469	0.17482	0.15638	0.414314	$\hat{\alpha }=6.06185\;\left(0.757291\right)$
										$\hat{\lambda }=0.0733611\;\left(0.0022748\right)$
ED	-48.6351	-95.2702	-95.1369	-93.8045	-94.7844	10.6955	2.28867	0.499509	<0.00001	$\hat{a}=12.4267\;\left(2.19674\right)$
LND	-48.7144	-95.4289	-95.2955	-93.9631	-94.943	10.6615	2.28014	0.498463	<0.00001	$\hat{\alpha }=13.2959\;\left(2.2062\right)$
LD	-48.6351	-93.2702	-92.8564	-90.3388	-92.2985	10.6955	2.28868	0.49951	<0.00001	$\hat{\lambda }=3.38536*10^{8}\;\left(1.11417*10^{1}2\right)$
										$\hat{\beta }=2.72426*10^{7}\;\left(8.96596*10^{1}0\right)$
FWD	-91.0456	-174.091	-172.61	-168.228	-172.148	1.13469	0.17482	0.15638	0.414314	$\hat{\alpha }=4.31329\;\left(2074.01\right)$
										$\hat{\beta }=0.00234378\;\left(5.36142\right)$
										$\hat{k}=1.40539\;\left(675.77\right)$
										$\hat{\lambda }=5.45624\;\left(8703.45\right)$
MD	-76.2751	-150.55	-150.417	-149.084	-150.064	5.58309	1.0925	0.326564	0.0021721	$\hat{\alpha }=226.359\;\left(32.6721\right)$

**Table 10:** Numerical values for analyzing the second COVID-19 real data set.

Model	−L	*I*_1_	*I*_2_	*I*_3_	*I*_4_	*F*_1_	*F*_2_	*F*_3_	*F*_3_(*p*)	Est. parameters (SEs)
WBHD	-61.4194	-118.839	-118.514	-115.461	-117.617	1.17518	0.199343	0.164772	0.227565	$\hat{a}=42.1679\;\left(17.8833\right)$
										$\hat{\alpha }=18.7576\;\left(2.4779\right)$
BHD	-22.748	-43.4961	-43.3908	-41.8072	-42.8854	11.2068	2.34283	0.429188	<0.00001	$\hat{a}=4.04395\;\left(0.770753\right)$
IPBHD	14.1059	32.2119	32.5362	35.5896	33.4332	35.7501	7.3048	0.781894	<0.00001	$\hat{\alpha }=0.0381204\;\left(0.0370304\right)$
										$\hat{\eta }=0.923866\;\left(0.118217\right)$
LBHED	-54.7254	-107.451	-107.345	-105.762	-106.84	44.2181	8.19748	0.737804	<0.00001	$\hat{\alpha }=6.86179\;\left(1.00323\right)$
APED	-60.6385	-117.277	-116.953	-113.899	-116.056	1.29451	0.205561	0.341714	0.197891	$\hat{\alpha }=2.01281\times 10^{10}\;\left(4.55112\times 10^{10}\right)$
										$\hat{a}=17.6897\;\left(1.03505\right)$
FD	-57.3966	-110.793	-110.469	-107.415	-109.572	1.84535	0.316168	0.183209	0.136365	$\hat{\alpha }=3.70919\;\left(0.418266\right)$
										$\hat{\lambda }=0.174655\;\left(0.00792412\right)$
ED	-23.363	-44.726	-44.6207	-43.0371	-44.1153	10.9023	2.26604	0.421709	<0.00001	$\hat{a}=4.87477\;\left(0.770769\right)$
LND	-23.8211	-45.6422	-45.537	-43.9534	-45.0316	10.7158	2.21921	0.417015	<0.00001	$\hat{\alpha }=5.61204\;\left(0.784427\right)$
LD	-23.363	-42.726	-42.4017	-39.3482	-41.5047	10.9023	2.26604	0.421709	<0.00001	$\hat{\lambda }=3.42662\times 10^{8}\;\left(1.03015\times 10^{1}2\right)$
										$\hat{\beta }=7.02928\times 10^{7}\;\left(2.11322\times 10^{1}1\right)$
FWD	-57.3966	-106.793	-105.65	-100.038	-104.351	1.84535	0.316168	0.183209	0.136365	$\hat{\alpha }=1.8593\;\left(2348.34\right)$
										$\hat{\beta }=0.000309393\;\left(2.19698\right)$
										$\hat{k}=1.99493\;\left(2519.65\right)$
										$\hat{\lambda }=10.0318\;\left(41423.3\right)$
MD	-54.8751	-107.75	-107.645	-106.061	-107.139	3.4986	0.62246	0.212103	0.0547007	$\hat{\alpha }=33.8783\;\left(4.37367\right)$

### 6.1 Concluding remarks on the application results

1. The WBHD is more flexible than our family baseline model (BHD) for fitting the two COVID-19 actual data sets.
2. For the two COVID-19 actual data sets that were taken into consideration for assessment, analytical measurements as well as MLE estimations and their accompanying standard errors (SE) are provided, respectively, in Tables 9 and 10, which provides us that our propose model is the best model for fitting the analyzed real data sets.
3. As a direct result of analyzing of the two COVID-19 actual data sets, we could arrive at the conclusion that the WBHD model performs far better than the other models that are comparable to it.

## 7 Conclusion

A new lifetime distribution, which was given the name WBHD, was presented in this paper. We derived its statistical properties. For the purpose of obtaining point estimates for the unknown WBHD parameters *α*, *a* eleven traditional estimation approaches were taken into consideration. A simulation research was carried out using R software, allowing for the comparison of the effectiveness of various estimating approaches. Two different sets of COVID-19 data were used to illustrate the benefits of the suggested distribution. In comparison to most of the other distributions under consideration, it was discovered that WBHD best suited the data. In addition to this, it can be shown in Figs 7 and 8 that the log-likelihood function has global maximum roots.

## 8 Future work

In the work that will be done in the future, we may make use of the model that was presented to model various actual data sets in a number of different areas, such as reliability engineering, survival analysis, and so on. Additionally, the WBHD may be expanded to include the introduction of bivariate WBHD’s, and this expansion can then be used to the modelling of actual data sets. It is possible to have a discussion about using Bayesian estimation to determine the suggested model parameters based on full and several kinds of censored samples and subjected to a variety of loss functions.

## References

[1] Shahzad U., Ahmad I., Almanjahie I., Hanif M., and Al-Noor N. H. L-moments and calibration based variance estimators under double stratified random sampling scheme: an application of covid-19 pandemic. Scientia Iranica, 2021. doi: 10.24200/sci.2021.56853.4942

[2] Lone S. A., Subzar M., and Sharma A. Enhanced estimators of population variance with the use of supplementary information in survey sampling. Mathematical Problems in Engineering, 2021, 2021. doi: 10.1155/2021/9931217

[3] Bhushan S., Kumar A., Akhtar M. T., and Lone S. A. Logarithmic type predictive estimators under simple random sampling. AIMS Mathematics, 7(7):11992–12010, 2022. doi: 10.3934/math.2022668

[4] Bhushan S., Kumar A., and Lone S. A. On some novel classes of estimators using ranked set sampling. Alexandria Engineering Journal, 61(7):5465–5474, 2022. doi: 10.1016/j.aej.2021.11.001

[5] Shahzad U., Ahmad I., Al-Noor N. H., and Benedict T. J. Use of calibration constraints and linear moments for variance estimation under stratified adaptive cluster sampling. Soft Computing, pages 1–12, 2022.

[6] Bhushan S., Kumar A., Shahzad U., Al-Omari A. I., and Almanjahie I. M. On some improved class of estimators by using stratified ranked set sampling. Mathematics, 10(18):3283, 2022. doi: 10.3390/math10183283

[7] Shahzad U., Ahmad I., Almanjahie I., and Al-Noor N. H. Utilizing l-moments and calibration method to estimate the variance based on covid-19 data. Fresenius Environmental Bulletin, 30(7A):8988–8994, 2021.

[8] Teamah A. A. M., Elbanna A. A., and Gemeay A. M. Fréchet-Weibull mixture distribution: Properties and applications. Applied Mathematical Sciences, 14(2):75–86, 2020. doi: 10.12988/ams.2020.912165

[9] Aljohani H. M., Almetwally E. M., Alghamdi A. S., and Hafez E. H. Ranked set sampling with application of modified kies exponential distribution. Alexandria Engineering Journal, 60(4):4041–4046, 2021. doi: 10.1016/j.aej.2021.02.043

[10] Wang W., Ahmad Z., Kharazmi O., Ampadu C. B., Hafez E. H., and Mohie El-Din M. M. New generalized- x family: Modeling the reliability engineering applications. Plos one, 16(3):e0248312, 2021. doi: 10.1371/journal.pone.0248312 33788850 PMC8011743

[11] Afify A. Z., Gemeay A. M., and Ibrahim N. A. The heavy-tailed exponential distribution: Risk measures, estimation, and application to actuarial data. Mathematics, 8(8):1276, 2020. doi: 10.3390/math8081276

[12] Abd El-Raheem A. M., Almetwally E. M., Mohamed M. S., and Hafez E. H. Accelerated life tests for modified kies exponential lifetime distribution: binomial removal, transformers turn insulation application and numerical results. AIMS Mathematics, 6(5):5222–5255, 2021. doi: 10.3934/math.2021310

[13] Almongy H. M., Almetwally E. M., Alharbi R., Alnagar D., Hafez E. H., and Mohie El-Din M. M. The weibull generalized exponential distribution with censored sample: estimation and application on real data. Complexity, 2021, 2021. doi: 10.1155/2021/6653534

[14] Tung Y. L., Ahmad Z., Kharazmi O., Ampadu C. B., Hafez E. H., and Mubarak Sh. A. M. On a new modification of the weibull model with classical and bayesian analysis. Complexity, 2021, 2021. doi: 10.1155/2021/5574112

[15] Teamah A. A. M., Elbanna A. A., and Gemeay A. M. Fréchet-Weibull distribution with applications to earthquakes data sets. Pakistan Journal of Statistics, 36(2), 2020.

[16] Teamah A. A. M., Elbanna A. A., and Gemeay A. M. Right truncated Fréchet-Weibull distribution: Statistical properties and application. Delta Journal of Science, 41(1):20–29, 2020.

[17] Almongy H. M., Almetwally E. M., Aljohani H. M., Alghamdi A. S., and Hafez E. H. A new extended rayleigh distribution with applications of covid-19 data. Results in Physics, 23:104012, 2021. doi: 10.1016/j.rinp.2021.104012 33728260 PMC7952137

[18] Al-Babtain A. A., Elbatal I., Al-Mofleh H., Gemeay A. M., Afify A. Z., and Sarg A. M. The flexible burr xg family: Properties, inference, and applications in engineering science. Symmetry, 13(3):474, 2021. doi: 10.3390/sym13030474

[19] Alfaer N. M., Gemeay A. M., Aljohani H. M., and Afify A. Z. The extended log-logistic distribution: Inference and actuarial applications. Mathematics, 9(12):1386, 2021. doi: 10.3390/math9121386

[20] Bakouch H., Chesneau C., and Enany M. A weighted general family of distributions: Theory and practice. Computational and Mathematical Methods, page e1135, 2020.

[21] Artzner P. Application of coherent risk measures to capital requirements in insurance. North Am. Actuar. J., 3(2):11–25, 1999. doi: 10.1080/10920277.1999.10595795

[22] Isaic-Maniu A. and Voda V. G. h. Generalized Burr-Hatke equation as generator of a homographic failure rate. Journal of applied quantitative methods, 3(3), 2008.

[23] Afify A. Z., Aljohani H. M., Alghamdi A. S., Gemeay A. M., and Sarg A. M. A new two-parameter burr-hatke distribution: Properties and bayesian and non-bayesian inference with applications. Journal of Mathematics, 2021, 2021. doi: 10.1155/2021/1061083

[24] Abouelmagd T. H. M. The logarithmic burr# hatke exponential distribution for mod# eling reliability and medical data. International Journal of Statistics and Probability, 7(5):1, 2018. doi: 10.5539/ijsp.v7n5p73

[25] Mahdavi A. and Kundu D. A new method for generating distributions with an application to exponential distribution. Communications in Statistics-Theory and Methods, 46(13):6543–6557, 2017. doi: 10.1080/03610926.2015.1130839

